# Marine antifouling biocide 4,5-dichloro-2-n-octyl-4-isothiazolin-3-one disrupts sediment microbiome structure and function: insights from absolute quantification and enzyme activity dynamics

**DOI:** 10.1128/aem.00819-26

**Published:** 2026-07-24

**Authors:** Jiaxin Zhang, Libing Cai, Lin Wang, Lianhui Zhang, Nan Meng, Andong Chen, Qiao Ma

**Affiliations:** 1 Institute of Environmental Systems Biology, College of Environmental Science and Engineering, Dalian Maritime University 12421, Dalian, China; University of Delaware, Lewes, Delaware, USA

**Keywords:** DCOIT, marine sediment, absolute quantification, microbial response, ecotoxicological risk

## Abstract

The organic booster biocide DCOIT (4,5-dichloro-2-n-octyl-4-isothiazolin-3-one) is widely used in marine antifouling systems, yet its ecological impacts on sediment microbiomes remain poorly understood. Here, we integrated absolute quantitative 16S rRNA gene sequencing, metagenomics, and enzyme activity assays to examine microbial responses to DCOIT exposure (0–50 μg/g sediment) over 30 days. DCOIT induced oxidative stress and bioenergetic impairment, accompanied by reduced microbial activity and inhibition of key enzyme-mediated processes involved in organic matter turnover and nitrogen transformation. Absolute quantification revealed a compensatory increase in total microbial abundance by Day 30, despite persistent diversity loss and community restructuring. Metagenomic analysis showed that DCOIT disturbed functional potentials related to carbon and nitrogen cycling. *Kordiimonas*, *Aliikangiella*, and *Neptuniibacter* emerged as potential contributors to nitrogen transformation, whereas *Marinobacter* was more closely associated with potential DCOIT transformation. DCOIT exposure also enriched adaptive traits, including chemotaxis, motility, quorum sensing, and biofilm regulation, and was accompanied by increased multidrug efflux systems and heavy metal resistance determinants. Our findings provide novel insights into the ecotoxicological risks of isothiazolinone biocides and highlight the potential for DCOIT to undermine sediment ecosystem functions and microbial habitat health. Given its extensive application, this study emphasizes the need to consider the microbial ecological consequences of DCOIT accumulation in seafloor environments.

**IMPORTANCE:**

DCOIT is widely used in marine antifouling coatings and can accumulate in benthic sediments, yet its effects on sediment microbiomes remain poorly defined. This study shows that DCOIT disrupts microbial energy status, enzyme activities, community structure, and nitrogen-cycling functions while selecting for adaptive traits and resistance-related determinants. By integrating absolute quantification, metagenomics, and enzyme assays, our work demonstrates that DCOIT poses microbial ecological risks beyond toxicity to macroorganisms and should be considered in assessments of antifouling biocides.

## INTRODUCTION

The rapid expansion of marine industries, including shipping, aquaculture, and offshore engineering, has intensified anthropogenic pressures on coastal and marine ecosystems (1). Among the major challenges associated with these activities is marine biofouling, which imposes substantial economic burdens through increased hydrodynamic drag, fuel consumption, maintenance requirements, and structural corrosion (2–4). Historically, organotin-based coatings, particularly tributyltin (TBT), dominated antifouling strategies. However, their environmental persistence and endocrine-disrupting effects led to a global ban on organotin-based antifouling systems under the International Convention on the Control of Harmful Anti-fouling Systems on Ships, which entered into force in 2008 (5). In the post-TBT era, 4,5-dichloro-2-n-octyl-4-isothiazolin-3-one (DCOIT), the active ingredient in SeaNine 211, has emerged as a widely used organic booster biocide (6). DCOIT was initially regarded as an effective and environmentally friendly alternative, as reflected by its recognition in the 1996 Presidential Green Chemistry Challenge Award (7). Although early studies suggested that DCOIT could undergo rapid degradation in natural seawater (8–10), accumulating evidence indicates that its environmental behavior and ecological effects are more complex than originally expected, raising concerns about its persistence, sediment accumulation, and potential risks to marine ecosystems (11–13).

Owing to its hydrophobicity (log K*ow* = 2.8–6.4), DCOIT rapidly partitions from the water column into benthic sediments, making sediments an important sink for this compound (11, 14–16). DCOIT has been frequently detected in coastal environments at concentrations ranging from ng/L to μg/L in water, with reported levels of 100 ng/L in Japan, 283 ng/L in Denmark, and 3,700 ng/L in Spanish coastal waters (17–20). Sediment surveys have further revealed its widespread occurrence, with concentrations ranging from <0.1 ng/g to over 1,600 ng/g across Asia, Europe, and South America (11), and a recent study recorded levels as high as 43,139 ng/g in Guanabara Bay (20). Such environmental occurrence is ecologically relevant because DCOIT and its transformation products can exert toxicity across multiple trophic levels (10). Acute and chronic effects have been documented in diverse aquatic organisms, including microalgae, mollusks, crustaceans, and teleost fish. For instance, Campos et al. reported an EC₅₀ of 8.3 μg/L for brown mussel larval development and an LC₅₀ of 0.5 μg/g for the amphipod *Tiburonella viscana* (19, 21, 22). Chen and co-workers demonstrated that DCOIT can bioaccumulate in medaka and act on molecular targets (e.g., thyroid hormone receptors), thereby posing multifaceted hazards to thyroid function, reproduction, development, and the nervous system (11–13, 23). Taken together, the documented occurrence, pseudo-persistence, and multi-trophic toxicity of DCOIT highlight the need to further evaluate its ecological risks in marine benthic environments (21, 23, 24).

Despite increasing evidence for the toxicity of DCOIT to aquatic macroorganisms, its impacts on sediment microbial communities remain insufficiently understood. This represents a critical knowledge gap because sediment microbiomes are key drivers of benthic ecosystem functioning, mediating organic matter mineralization and nutrient cycling (25). Given that DCOIT is designed as a broad-spectrum biocide and tends to accumulate in sediments, benthic microbial communities are likely to experience direct and prolonged exposure (16). However, their sensitivity, structural responses, and functional resilience under DCOIT stress remain poorly characterized, limiting a comprehensive assessment of how modern antifouling biocides affect sediment ecosystem services.

To address these gaps, we established sediment microcosms across a DCOIT concentration gradient of 0, 0.005, 0.5, and 50 μg/g sediment. By integrating enzyme activity assays, absolute quantitative 16S rRNA gene sequencing, and metagenomic analysis, this study aimed to (i) evaluate the impacts of DCOIT on microbial activity and bioenergetic homeostasis; (ii) elucidate the shifts in microbial abundance, diversity, and community structure; and (iii) characterize the metagenome-resolved functional responses of sediment microbial communities, including changes in biogeochemical nutrient cycling, stress adaptation, and resistance-related determinants. The application of absolute quantification further enabled us to overcome the inherent limitations of conventional relative-abundance-based metrics, which often mask actual fluctuations in microbial abundance under chemical stress (26). These findings advance our understanding of DCOIT’s ecological effects at the microbial level and provide a basis for the environmental risk assessment and sustainable management of marine benthic habitats.

## RESULTS AND DISCUSSION

### DCOIT disrupts microbial metabolism and key enzyme activities

**Fig 1:**
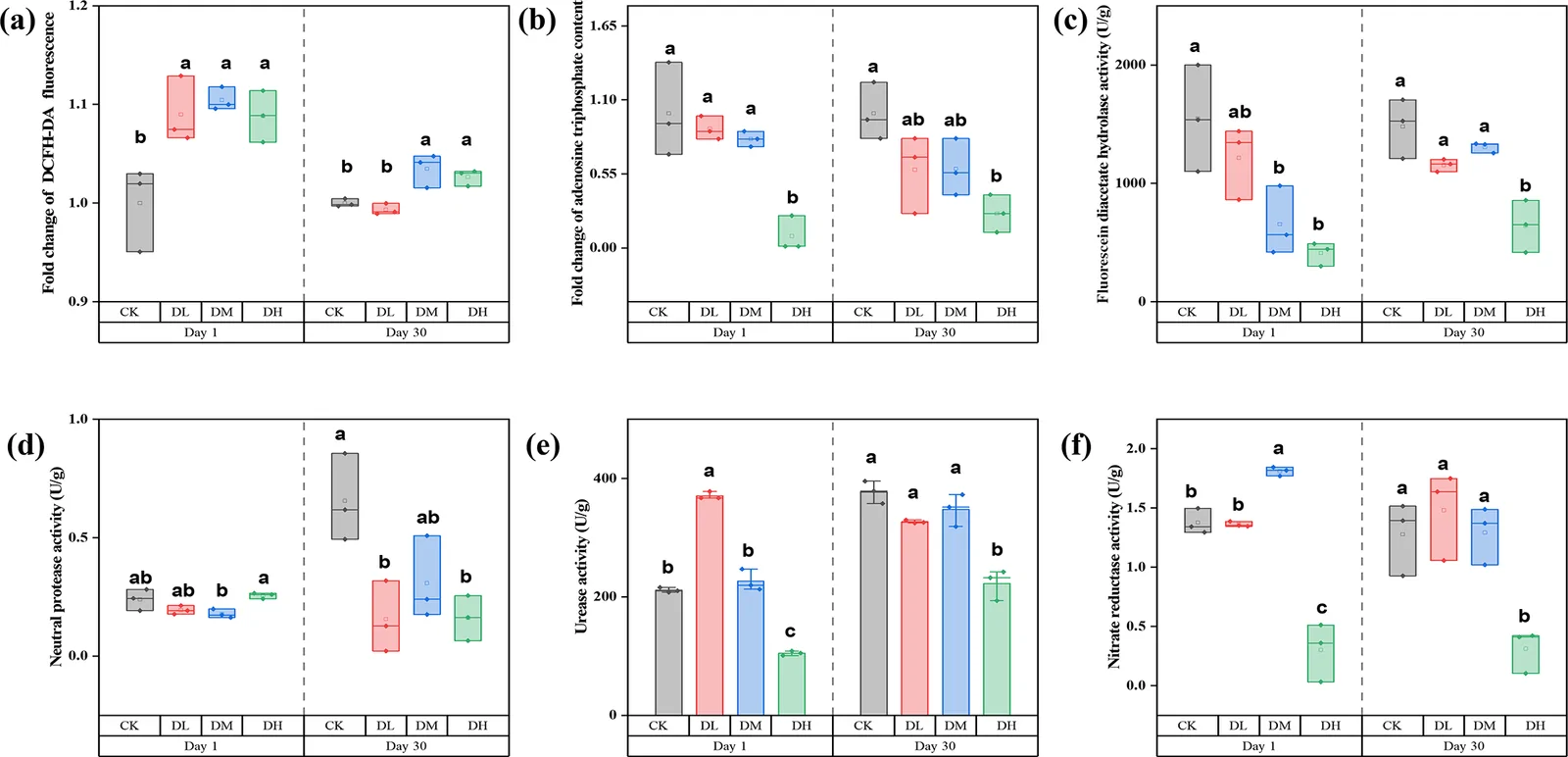
Impact of DCOIT on microbial metabolic indicators and key enzyme activities in marine sediment microcosms. (**a**) ROS production, (**b**) ATP content, (**c**) FDA hydrolase activity, (**d**) NP activity, (**e**) UE activity, and (**f**) NR activity. Different lowercase letters above the boxes indicate significant differences among treatment groups within the same sampling time, whereas shared letters indicate no significant difference.

The experimental workflow is shown in Fig. S1, with metabolic and enzymatic indicators assessed on Days 1 and 30. DCOIT exposure triggered a significant oxidative stress response in the marine sediment microbial community (Fig. 1a). Upon acute exposure on Day 1, reactive oxygen species (ROS) levels were significantly elevated in all treatment groups, showing 1.09-, 1.10-, and 1.09-fold increases in the DL, DM, and DH groups relative to the control, respectively. Despite these ROS changes, catalase (CAT) activity remained unaffected on both Day 1 and Day 30 (Fig. S2). The oxidative stress response was accompanied by a decline in microbial energy status, particularly under high-dose DCOIT exposure. ATP content in the DH group decreased by 91.20% and 74.38% relative to the control on Day 1 and Day 30, respectively, indicating severe bioenergetic impairment (Fig. 1b). Consistently, fluorescein diacetate (FDA) hydrolase activity decreased by 50.36% and 68.79% in the DM and DH groups on Day 1, respectively (Fig. 1c). Although the DM group exhibited a partial recovery by Day 30, the DH group remained significantly inhibited, with activity levels 53.06% lower than that of the control. These results indicated that high-dose DCOIT exposure caused persistent bioenergetic impairment and reduced the overall metabolic activity of the marine sediment microbiome (19, 27, 28).

To assess whether DCOIT altered sediment nutrient-cycling capacity, we measured key enzymes involved in organic matter decomposition and nitrogen transformation. Neutral protease (NP) activity, associated with protein degradation and C–N coupling (29), was largely unchanged on Day 1 but decreased by 76.26% and 75.40% in the DL and DH groups, respectively, by Day 30 (Fig. 1d). Urease (UE), which hydrolyzes urea to ammonia (30), decreased in the DH group by 55.57% on Day 1 and 43.04% on Day 30 (Fig. 1e). Nitrate reductase (NR), which mediates nitrate reduction to nitrite (31), was also inhibited in the DH group, decreasing by 59.33% and 70.04% on Day 1 and Day 30, respectively (Fig. 1f). By Day 30, UE and NR activities were not significantly affected in the DL or DM groups. These results suggested that high-dose DCOIT selectively impaired key enzymatic processes involved in nitrogen transformation. Consistently, NH_4_^+^ concentrations decreased in DCOIT-treated sediments (Fig. S3), which may reflect reduced inorganic nitrogen regeneration associated with suppressed microbial metabolic activity and nitrogen-transforming enzymes (26). In contrast, polyphenol oxidase (PPO) activity, involved in the oxidation of recalcitrant organic compounds and humification, increased by 1.19-, 1.24-, and 1.11-fold in the DL, DM, and DH groups, respectively (Fig. S4), suggesting a distinct oxidative-enzyme response rather than uniform suppression across all cellular functions.

### DCOIT affects microbial abundance and diversity

Relative-abundance-based profiling can mask actual changes in total microbial abundance under chemical stress (32). Therefore, we employed absolute quantitative 16S rRNA gene sequencing (Accu16S) to assess community dynamics. Comparative analysis revealed clear discrepancies between the two methods. Absolute quantification revealed additional genus-level signals that were not captured by relative-abundance-based differential analysis, and among the 471 genera identified as differentially abundant by absolute quantification, 52 were not detected as differentially abundant by relative profiling (Fig. S5). In the DH group, total microbial abundance initially decreased by 16.57% on Day 1 but increased to 1.41-fold of the control level by Day 30 (Table S1). This biphasic pattern suggests an initial toxic shock followed by compensatory microbial proliferation. Such temporal plasticity may reflect multilevel adaptive responses, including the selection of DCOIT-tolerant taxa, enrichment of potential degraders, and reorganization of community structure (33).

**Fig 2:**
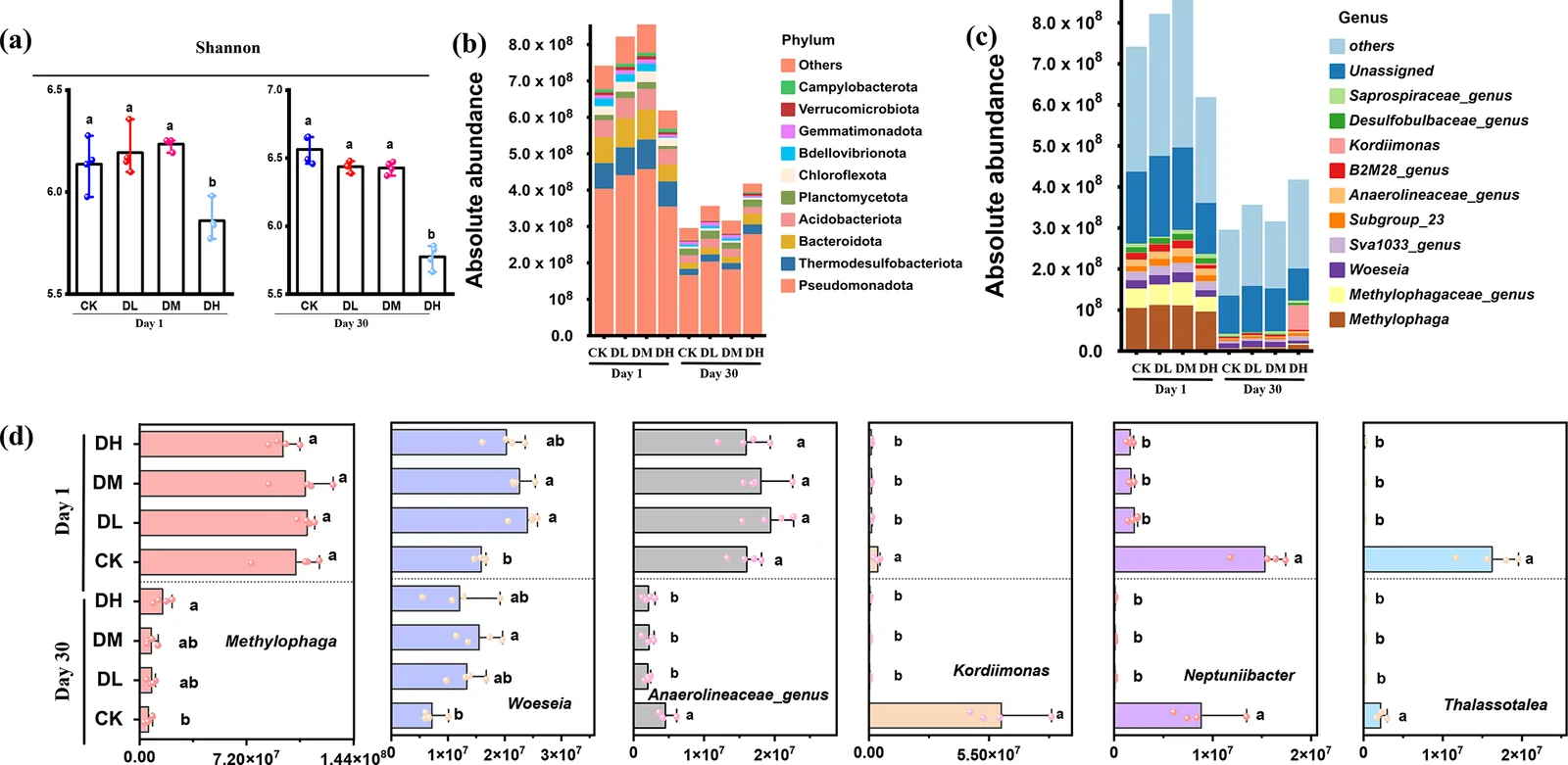
Effects of DCOIT on α-diversity and taxonomic composition of the marine sediment microbial community. (**a**) Shannon index; bacterial community composition at the (**b**) phylum and (**c**) genus levels; (**d**) absolute abundances of the predominant genera.

Partial least squares discriminant analysis (PLS-DA) revealed shifts in bacterial community structure (Fig. S6). The DH group clustered separately from all other groups at both time points, whereas the DL and DM groups diverged from the control on Day 1 but converged by Day 30. High-dose DCOIT exposure was also associated with a persistent reduction in microbial diversity (Fig. 2a and Table S2). Specifically, the Shannon index decreased from 6.14 to 6.02 on Day 1 and from 6.54 to 5.78 on Day 30. Faith’s PD in the DH group decreased by 13.06% (Day 1) and 31.31% (Day 30) relative to the control (Fig. S7). These results indicated sustained toxic effects of high-dose DCOIT, characterized by progressive diversity loss rather than community recovery.

### DCOIT reshapes microbial community structure

Absolute quantitative taxonomic profiling identified 15,860 amplicon sequence variants (ASVs) distributed across 70 phyla. The dominant bacterial phyla based on absolute and relative quantification are shown in Fig. 2b and Fig. S8, respectively. The microbial community was dominated by Pseudomonadota (2.49 × 10^9^ copies/g, 56.25%), Bacteroidota (3.62 × 10^8^, 8.18%), Thermodesulfobacteriota (3.78 × 10^8^, 8.54%), Acidobacteriota (2.87 × 10^8^, 6.50%), and Planctomycetota (1.37 × 10^8^, 3.10%). After 30 days of exposure, the absolute abundances of Pseudomonadota, Bacteroidota, and Thermodesulfobacteriota in the DH group increased by 1.68-, 1.56-, and 1.79-fold, respectively, relative to the CK group.

At the genus level, 894 taxa were identified. The major genera included *Methylophaga*, *Methylophagaceae_genus, Woeseia*, *Subgroup_23*, and *Anaerolineaceae_genus*, contributing 10.47%, 4.41%, 2.96%, 1.88% and 1.81% (Fig. 2c and Fig. S9). DCOIT exposure induced concentration- and time-dependent shifts in several responsive genera (Fig. 2d and Fig. S10). *Neptuniibacter* and *Thalassotalea* were rapidly enriched under high-dose exposure, with abundances 9.32- and 263.46-fold higher than the control on Day 1 and increasing further to 104.25- and 535.17-fold higher by Day 30, respectively. *Kordiimonas* also showed a profound 134.96-fold increase in absolute abundance in the DH group by Day 30. Conversely, *Saprospiraceae_genus* was suppressed, decreasing by 61.05% on Day 1 and 83.91% by Day 30. *B2M28_genus* decreased by 47.39% in the DH group on Day 1 but recovered on Day 30.

A 3-month selective acclimation of the DH sediment yielded a stable microbial consortium that achieved complete removal of 20 mg/L DCOIT within 48 h (Fig. S11). This consortium was characterized by the enrichment of *Mycobacterium* (9.63%) and *Marinobacter* (8.43%), followed by *Sutcliffiella*, *Rhodanobacter*, and *Rhizomicrobium* (Fig. S12). These enriched taxa may represent potential contributors to DCOIT tolerance and biotransformation in the acclimated consortium.

### Metagenomic insights into functional reconfiguration and stress adaptation

To characterize the functional reconfiguration of the sediment microbiome under DCOIT stress, absolute metagenomic sequencing was performed on Day 30. A total of 76.5 GB of clean data (515,205,528 reads) was generated and assembled into 2,159,562 non-redundant contigs (≥500 bp). Among the 7,625 KEGG orthologs (KOs) annotated in the Kyoto Encyclopedia of Genes and Genomes (KEGG) database, 1,435 KOs were differentially abundant in the DH group compared to the control (Fig. S13). Specifically, 1,046 KOs increased in abundance, and 389 KOs decreased.

**Fig 3:**
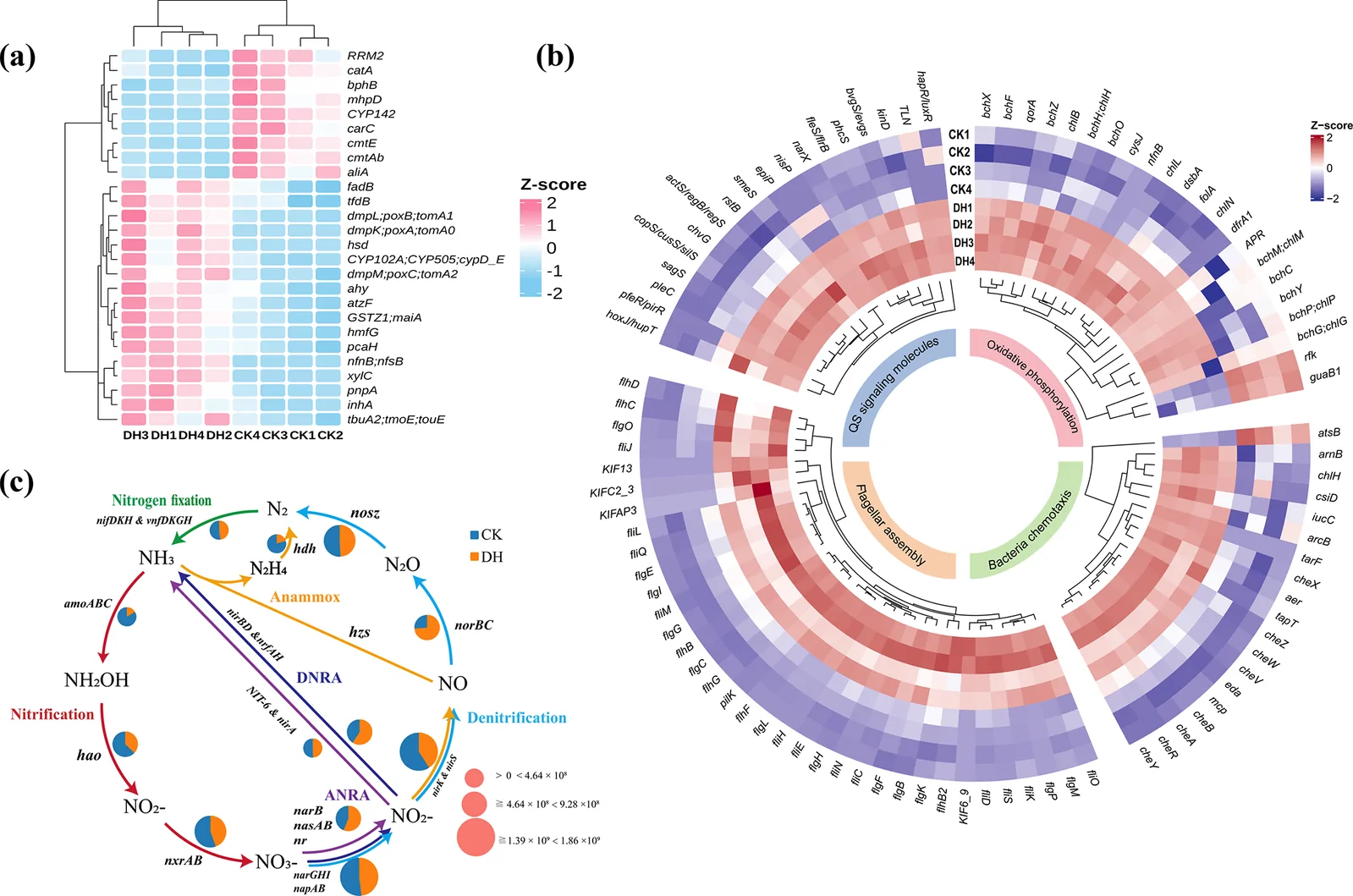
Metagenomic insights into functional reconfiguration and adaptive responses under DCOIT stress. (**a**) Heatmap showing the abundance patterns of differentially abundant KO features associated with xenobiotic biodegradation and metabolism. (**b**) Circular heatmap showing genes related to adaptive pathways, including bacterial chemotaxis, motility, quorum sensing, and oxidative phosphorylation. (**c**) Schematic overview of DCOIT-induced changes in nitrogen-cycling pathways based on NCycDB-annotated genes.

KEGG annotation identified 305 genes within the “Xenobiotics biodegradation and metabolism” category (Level 2), among which 26 were differentially abundant, including 17 enriched and 9 depleted genes. As shown in Fig. 3a, DCOIT exposure enriched multiple genes associated with aromatic compound metabolism, including *tbuA2* (toluene monooxygenase, FC = 2.04), *pcaH* (protocatechuate 3,4-dioxygenase, FC = 2.12), *dmp* family genes involved in phenol degradation (FC = 2.28–249.37), *xylC* (benzaldehyde dehydrogenase, FC = 81.93), *hsd* (hydroxysteroid dehydrogenase, FC = 112.46), and CYP102A gene (cytochrome P450 monooxygenase, FC = 4.92). The enrichment of these genes suggested an enhanced functional potential for xenobiotic oxidation, aromatic transformation, and ring-cleavage processes under DCOIT stress (34).

Genes involved in redox cycling and stress response, including NADPH oxidoreductase (*qorA*, FC = 4.73), nitroreductases (*nfsB/nfnB*, not detected in the control), and sulfate reduction components (*APR*, FC = 19.23; *cysJ*, not detected in the control), were enriched under DCOIT exposure. In contrast, genes related to NADPH-dependent purine interconversion (*guaB1*, FC = 0.12; *GMPR*, FC = 0.41; *guaC*, FC = 0.41) and riboflavin kinase (*rfk*, FC = 0.04) decreased in abundance. These changes were consistent with the oxidative stress and bioenergetic impairment observed in the enzyme and ATP assays, suggesting that DCOIT disturbed microbial redox homeostasis and reducing-power metabolism.

DCOIT exposure further enriched genes associated with chemotaxis, motility, quorum sensing, and biofilm-related regulation (Fig. 3b). The chemotaxis gene cluster *cheBWRXYZ* (FC = 2.00–3.44), methyl-accepting chemotaxis proteins (*mcp*, FC = 2.99), and the aerotaxis receptor *aer* (FC = 4.65) increased in abundance, indicating enhanced potential for environmental sensing (35). Flagellar assembly and motility genes, including *fliC* (FC = 3.33), *fliD* (FC = 3.92), *flgBCFL* (FC = 2.42–7.05), *flhBCF* (FC = 2.18–5.24), *fliGMN* (FC = 1.81–3.70), and *motAB* (FC = 2.52–3.58), were also enriched (33). In addition, genes associated with quorum sensing, signal transduction, and biofilm-related regulation, such as *luxR* (FC = 309.79), *bvgS* (FC = 22.89), *sagS* (FC = 8.05), *traI* (FC = 6.31), *nisP* (FC = 225.25), *flrB* (FC = 6.09), and *qseB* (FC = 3.56), showed increased abundance (36). These responses suggested that the sediment microbiome may enhance environmental sensing and motility to locate less stressful microhabitats or favorable niches under DCOIT pressure (37). The concurrent enrichment of quorum-sensing and biofilm-related genes further implied a shift toward coordinated community-level adaptation, potentially improving collective tolerance to DCOIT-induced oxidative and metabolic stress (38).

### DCOIT enriches resistance-related functional genes

Functional profiling against the BacMet database revealed that DCOIT exposure triggered the enrichment of resistance-related genes in the sediment microbiome (Fig. S14). Multidrug efflux pump determinants, including *pmrA* and *vmeJ,* emerged exclusively under high-dose stress. Concomitantly, several heavy metal resistance-related categories were enriched, including the functional traits conferring resistance to gold (FC = 13.63), mercury (FC = 2.62), and phenylmercury acetate (FC = 2.33) (Fig. S15). This pattern was further supported by the enrichment of the *mer* operon (FC = 4.28–130.66) and gold resistance-related genes, including *golT* (FC = 13.33) and the *gesABC* efflux system (FC = 11.03–214.82) (Fig. S14) (39, 40). The *gesABC* genes are capable of extruding heavy metals, antibiotics, and biocides (41). This synergistic activation of biocide efflux and metal detoxification systems suggested that DCOIT exposure may select for microbial traits linked to multidrug and heavy metal resistance. Although further evidence is needed to confirm direct co-selection mechanisms, the enrichment of these resistance determinants indicates a potential ecological risk of DCOIT in expanding resistance-related functional reservoirs in marine sediments.

### DCOIT alters the biogeochemical cycling of carbon and nitrogen

The abundances of key nodes within the canonical glycolysis and tricarboxylic acid (TCA) cycles remained relatively stable, indicating that the core carbon metabolic skeleton of the microbiome was not broadly disrupted (Fig. S16). However, the Entner-Doudoroff (ED) pathway, an alternative glycolytic route with lower energy yield but reduced enzymatic protein investment (42), increased in the DH group (FC = 1.63). This shift may represent a metabolic adjustment toward lower-cost carbohydrate utilization under DCOIT stress. Furthermore, genes related to pyruvate conversion showed altered abundance patterns. The anaplerotic conversion of pyruvate to oxaloacetate, which replenishes the TCA cycle (43), exhibited the highest baseline abundance in the control (4.53 × 10^9^ copies/g) but was lower in the DH group (4.14 × 10^9^ copies/g). In contrast, the genetic potential for converting pyruvate to lactate and acetate increased by 2.05- and 1.25-fold, respectively, and the pathway from lactate to acetate increased by 1.38-fold. These results suggested that DCOIT exposure may redirect part of the microbial carbon metabolic potential toward fermentative pathways.

Based on NCycDB annotation, 12 out of the 58 identified nitrogen-cycling genes were differentially abundant under DCOIT exposure (Fig. 3c). Notably, genes encoding ammonia monooxygenase subunits (amoABC; FC = 0.07–0.52), which mediate the first step of nitrification, were strongly reduced (44). The *hzo* gene (FC = 0.25), an anammox-associated hydrazine oxidation gene, also decreased in abundance. These patterns indicated reduced genetic potential for ammonia oxidation and anammox-related nitrogen transformation. In contrast, genes associated with nitrate reductase systems (*napBC*, FC = 2.35–4.40; *narJW*, FC = 2.53–ND) and nitric oxide reductases (*norBC*, FC = 2.20–2.35), involved in nitrate/nitrite reduction and NO reduction, respectively, were enriched (45). This suggested an increased metagenomic potential for downstream nitrate reduction and denitrification-related processes. However, NR enzyme activity was significantly inhibited in the DH group (Fig. 1f), indicating a mismatch between gene abundance and enzyme activity. This discrepancy may be associated with DCOIT-induced bioenergetic impairment and reduced overall microbial metabolic activity (Fig. 1a through c).

### Identification of key nitrogen-cycling taxa and functional gaps

**Fig 4:**
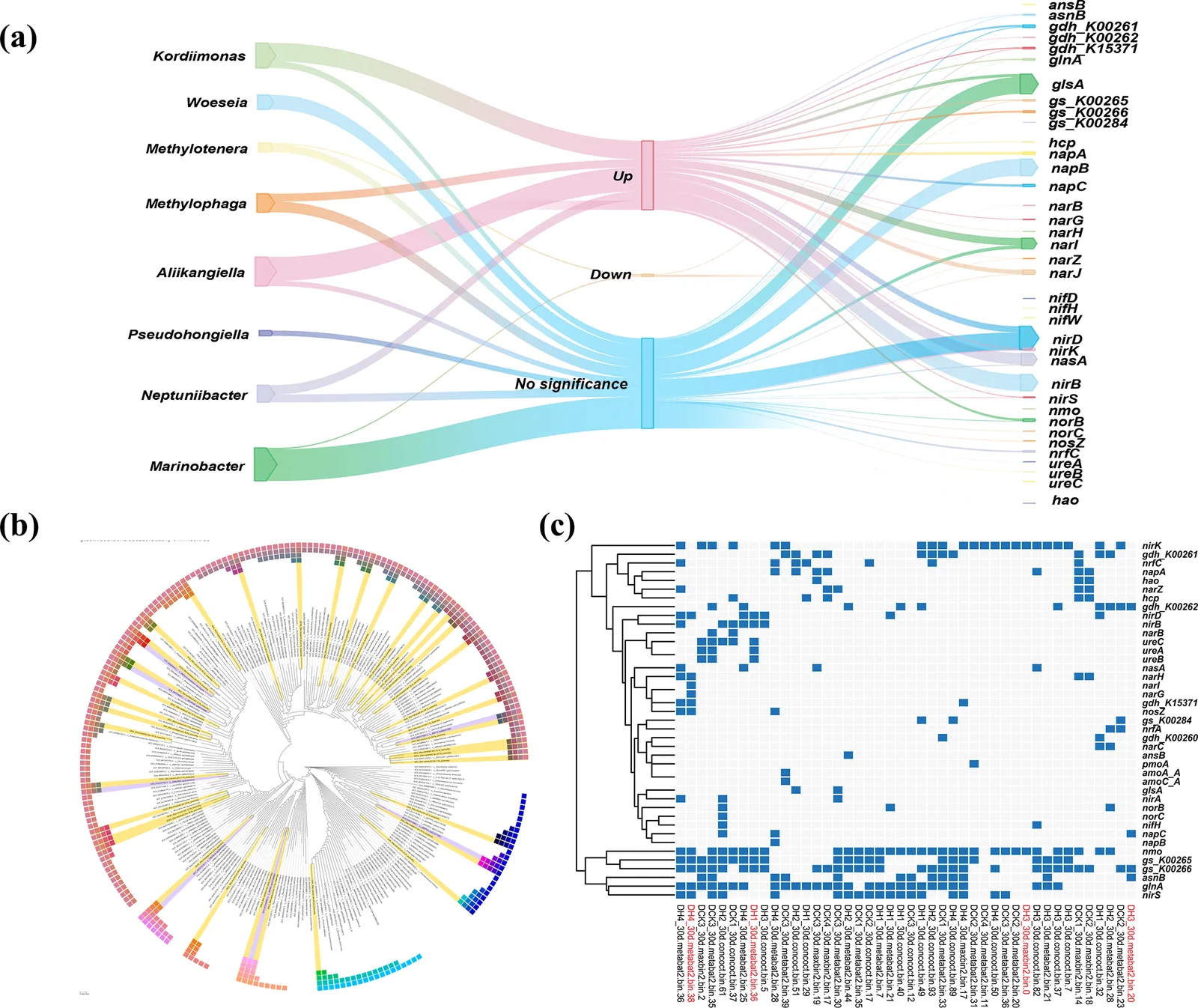
Genome-resolved evidence for nitrogen-cycling potential among key microbial populations. (**a**) Taxonomic contributions of abundant genera to nitrogen metabolism; (**b**) phylogenetic placement of the recovered MAGs within the GTDB reference tree; and (**c**) presence/absence heatmap of key nitrogen-cycling functional genes in the recovered MAGs.

To identify taxa potentially contributing to nitrogen metabolism under DCOIT stress, we performed a taxonomic contribution analysis of abundant bacterial genera based on NCycDB annotation (Fig. 4a). Several high-abundance taxa, such as *Methylotenera*, *Pseudohongiella*, and *Marinobacter*, showed limited contributions to nitrogen-cycling genes. In contrast, *Kordiimonas*, *Aliikangiella*, and *Neptuniibacter* were identified as major contributors to nitrogen transformation potential (Table S3). Specifically, *Kordiimonas* was mainly associated with *narGHI*, *nirKS*, *nirBD*, and *asnB* genes. *Aliikangiella* contributed to *napAC*, *narGHIJZ*, *nirB*, *nirKS*, *hcp*, and *asnB*, whereas *Neptuniibacter* was associated with *norB*, *nirBK*, and *nasA*. Notably, these taxa primarily contributed to nitrate/nitrite conversion pathways rather than ammonia oxidation. Further analysis showed that *amoABC* genes were mainly assigned to typical ammonia-oxidizing taxa, including *Nitrosopumilus*, *Nitrosomonas*, and *Nitrosospira* (Table S4), which occurred at relatively low abundance in the sediment community.

To provide genome-resolved evidence for the inferred functional roles, metagenomic binning recovered 44 metagenome-assembled genomes (MAGs), 36 of which were shown in Table S5 with completeness >70% and contamination <10%. These MAGs spanned several major taxonomic lineages, including Alphaproteobacteria, Gammaproteobacteria, Deltaproteobacteria, and Bacteroidota (Fig. 4b). Representative MAGs affiliated with *Kordiimonas*, *Aliikangiella*, and *Neptuniibacter* were reconstructed, enabling further examination of their nitrogen-cycling potential. *Kordiimonas* genomes harbored genes covering the major steps of denitrification, including *narGHI*, *nirKS*, *norB*, and *nosZ* (Fig. 4c). The *Aliikangiella* MAG possessed *napC*, alongside *gdh*, *asnB*, and *gltB*, suggesting potential involvement in electron transfer associated with nitrate reduction. *Neptuniibacter* MAG contained *narH* and *nirBD*, indicating potential for dissimilatory nitrate reduction to ammonium, complemented by *nmo* and *gdh* for broader nitrogen transformation. Collectively, these genome-resolved results supported the taxonomic contribution analysis and revealed niche differentiation among key nitrogen-cycling taxa.

### Integrated impacts of DCOIT on the marine sediment microbiome

Previous ecotoxicological assessments of DCOIT have mainly focused on its acute and chronic toxicity to aquatic macroorganisms, whereas its impacts on sediment microbial communities, the fundamental drivers of benthic biogeochemical cycles, remain poorly understood (10, 16). By integrating absolute quantitative 16S rRNA gene sequencing, metagenomics, and enzyme activity profiling, this study provides a multi-level view of how DCOIT perturbs marine sediment microbiomes. Overall, our results suggest that DCOIT acts as a multi-tiered microbial stressor, inducing oxidative stress and bioenergetic impairment, restructuring microbial communities, disrupting nutrient-cycling functions, and selecting for adaptive and resistance-related traits (Fig. S17). These linked responses indicate that DCOIT may compromise not only microbial community composition but also the functional stability of sediment ecosystems.

DCOIT exposure induced oxidative stress and bioenergetic impairment, as evidenced by increased ROS levels, ATP depletion, and inhibited FDA hydrolase activity, particularly under high-dose exposure (Fig. 1). This physiological disturbance is consistent with the known biocidal action of isothiazolinones, which involves the disruption of cellular redox homeostasis through thiol depletion and redox imbalance (46). Notably, although DCOIT was introduced as a single spike and may have been degraded during incubation (8, 10), its impacts persisted across the 30-day experiment. This implied a potential legacy effect, whereby the initial shock altered the subsequent trajectory of community succession. The absolute abundance data further revealed a biphasic response, with an early decrease followed by an increase in microbial abundance by Day 30 (Table S1). This pattern suggests partial compensatory proliferation of tolerant or potentially DCOIT-transforming populations rather than simple community recovery. In natural marine environments, DCOIT is continuously released from antifouling coatings into surrounding waters and sediments (16, 21, 22). Such chronic exposure may impose more sustained selective pressure than the single-dose microcosm used here, highlighting the need for future continuous-flow or repeated-dosing experiments to better simulate field conditions.

High-dose DCOIT caused pronounced community restructuring and functional disorder in the sediment microbiome (Fig. 2). Importantly, the increase in microbial abundance by Day 30 did not indicate functional recovery but was accompanied by diversity loss and an imbalance in nitrogen-cycling potential. The genetic potential for nitrification was constrained, as *amoABC* was mainly associated with low-abundance ammonia-oxidizing taxa (Table S4), whereas nitrate/nitrite reduction potential was concentrated in enriched *Kordiimonas*, *Aliikangiella*, and *Neptuniibacter*, which harbored abundant *nar*/*nir*/*nor*-related genes (Fig. 4). By contrast, several background taxa, including *Woeseia*, *Methylophaga*, and *Pseudohongiella*, contributed little to nitrogen-cycling genes, indicating that DCOIT selectively reshaped the functional roles of specific populations rather than uniformly altering the whole community. Although *Marinobacter* was enriched, its nitrogen-cycling gene contribution changed little (Fig. S12; Fig. 4); together with its known aromatic-degradation capacity, this pattern suggests a closer association with DCOIT transformation than with nitrogen cycling (47). More importantly, the enrichment of nitrate-reduction and denitrification-related genes contrasted with the inhibition of nitrate reductase activity, revealing a genotype–phenotype decoupling in nitrogen transformation. We speculate that, under DCOIT-induced bioenergetic stress, microbial resources may be preferentially allocated to survival-related processes, such as oxidative stress defense, efflux-mediated protection, and xenobiotic transformation, thereby limiting the realization of nitrogen-cycling gene potential at the enzymatic level. This decoupling suggests that metagenomic functional potential alone may overestimate realized ecosystem functioning under biocidal stress, and future metatranscriptomic and metaproteomic analyses are needed to clarify how DCOIT-induced community adaptation translates into sediment biogeochemical activity.

DCOIT-induced selective pressure also selected for functional traits associated with stress adaptation and resistance-related risks. The enrichment of genes involved in xenobiotic transformation, chemotaxis, flagellar motility, quorum sensing, and biofilm-related regulation suggests that the sediment microbiome may enhance environmental sensing, niche exploration, cell–cell communication, and community-level protection under DCOIT stress (Fig. 3b). Such adaptive responses may facilitate survival under oxidative and metabolic stress and contribute to the enrichment of tolerant or potentially DCOIT-transforming taxa (48). In parallel, the increased abundance of multidrug efflux systems and heavy metal resistance determinants, including gold- and mercury-resistant genes (Table S6 and Fig. S15), suggests an additional ecological risk associated with antifouling biocide exposure. Metagenomic localization of *mer* and *ges* clusters in enriched taxa, including *Marinobacter* and *Neptuniibacter*, further indicates a potential link between DCOIT-selected populations and cross-resistance traits (Table S7). Although direct co-selection mechanisms require further validation, these patterns suggest that DCOIT may expand resistance-related functional reservoirs in marine sediments (49).

Collectively, although DCOIT is widely promoted as a new-generation antifouling agent, its recognized aquatic toxicity and the microbial responses observed here indicate broader ecological implications for marine benthic systems. By linking physiological toxicity, bioenergetic impairment-driven functional decoupling, microbial adaptation, and resistance-related risks, this study provides new insights into the complex ecological pressures imposed by modern antifouling biocides in marine sediments.

### Conclusion

This study demonstrates that DCOIT exposure imposes multi-level disturbances on marine sediment microbiomes. DCOIT-induced oxidative stress and bioenergetic impairment reduced overall microbial metabolic activity and inhibited key enzyme-mediated processes involved in organic matter turnover and nitrogen transformation. Although absolute quantification revealed a compensatory increase in total microbial abundance by Day 30, this response was accompanied by persistent diversity loss, community restructuring, and a mismatch between nitrogen-cycling gene potential and nitrate reductase activity. Metagenomic analysis further showed that DCOIT selected for functional traits associated with xenobiotic transformation, stress adaptation, quorum sensing, biofilm regulation, and resistance-related determinants. Collectively, these findings indicate that DCOIT may pose long-term risks to the functional stability and ecological security of benthic ecosystems, highlighting the need to consider its microbial ecological risks in future environmental assessments and management of antifouling biocides.

## MATERIALS AND METHODS

### Chemicals and experimental design

DCOIT (purity >99%; Aladdin, China) was dissolved in dimethyl sulfoxide (DMSO) to prepare stock solutions. Marine sediment was collected from Xingshutun Pier (Dalian, China), washed three times with filtered natural seawater, and acclimated for 3 days (25°C, 150 rpm). Sediment microcosms were established in Erlenmeyer flasks containing 20 g (wet weight) of pretreated sediment and natural seawater (total volume 100 mL). Four treatments were established: control (CK, 0 μg/g) and low-, medium-, and high-dose DCOIT treatments, designated DL (0.005 μg/g), DM (0.5 μg/g), and DH (50 μg/g), respectively. The same volume of DMSO was added to the control and all treatment microcosms. All microcosms were incubated at 25°C and 150 rpm for 30 days in quadruplicate (Fig. S1). Samples were collected on Day 1 and Day 30 to represent short-term and longer-term exposure responses, respectively.

### Quantification of DCOIT

DCOIT concentrations in the acclimated consortium cultures were determined by ultra-performance liquid chromatography (UPLC; Waters ACQUITY, USA) (50, 51). Samples were mixed with an equal volume of methanol and filtered through a 0.22 μm membrane. Separation was performed using a water:methanol mobile phase (10:90, vol/vol) at 0.8 mL/min, with the column temperature set at 40°C and detection at 280 nm. DCOIT eluted at approximately 5.22 min.

### Determination of microcosm properties and enzyme activities

Ammonium nitrogen (NH_4_^+^-N) concentrations were determined spectrophotometrically using Nessler’s reagent. ROS levels were measured using a fluorescent probe-based assay kit (Abbkine, China), and adenosine triphosphate (ATP) content was quantified using a bioluminescence assay kit (Nanjing Jiancheng, China). Activities of NP, PPO, CAT, NR, UE, and FDA hydrolase were measured using commercial assay kits (Solarbio, China) according to the manufacturers’ instructions. Enzyme activity data are presented from three biological replicates (52, 53).

### Absolute quantitative 16S rRNA gene sequencing (Accu16S)

The absolute abundance of the bacterial 16S rRNA genes was determined using the Accu16S method (GeneSky, China). The V4 region was amplified with primers 515F/806R with adapter sequences. Synthetic spike-in standards were added to calculate absolute microbial abundance (copies/g sediment). The resulting libraries were sequenced on the Illumina platform. Bioinformatic analysis was performed using the QIIME2 pipeline (54). Raw sequences were quality-filtered, denoised, merged, and chimera-checked to generate ASVs. Taxonomic assignment was performed against the SILVA database (version 138.2). Absolute microbial abundance was calculated based on the linear relationship between the spike-in standards and their recovered reads (55).

### Absolute quantitative metagenomic sequencing

Absolute quantitative metagenomic sequencing was performed by GeneSky Biotechnology Co., Ltd. (China). Briefly, genomic DNA was extracted, and its integrity and concentration were assessed by agarose gel electrophoresis and Qubit fluorometric quantification. For absolute quantification, qualified DNA samples were spiked with internal standard sequences and fragmented to approximately 400 bp using a Covaris ME220 focused ultrasonicator. Sequencing libraries were constructed through end repair, 3′-adenylation, and ligation with Illumina adapters, followed by quality control and sequencing on the Illumina platform (56). The absolute abundance of functional genes was calculated based on the ratio of reads recovered from the internal standards to their initial spike-in concentrations. Functional annotation was performed against the KEGG, Antibacterial Biocide and Metal Resistance Genes Database (BacMet), and the Nitrogen Cycle Database (NCycDB). Metagenomic binning was conducted using MetaBAT2, MaxBin2, and CONCOCT within the MetaWRAP pipeline, followed by bin refinement using DAS Tool. Quality-filtered MAGs were uploaded to KBase for GTDB-Tk taxonomic profiling and phylogenomic tree reconstruction.

### Statistical analysis and data visualization

Data management and statistical analyses were mainly performed using Microsoft Excel 2021 and OriginPro 2021. Differences in microbial abundance, gene copies, enzyme activities, and physicochemical parameters among treatment groups were evaluated using one-way analysis of variance (ANOVA) or Student’s t-test where appropriate. Statistical significance was defined as *P* < 0.05, and differentially abundant metagenomic genes were identified using |log_2_ fold change| ≥ 1 and adjusted *P* < 0.1. Data visualization was performed using R (v.4.5.3) using RStudio (v.2025.09.2). Additionally, the online platform ChiPlot (https://www.chiplot.online) was also employed for graphical refinements. Data are presented as the mean ± standard deviation of biological replicates unless otherwise stated.

## Data Availability

The raw sequence data have been uploaded to the Genome Sequence Archive of the BIG Data Center (https://bigd.big.ac.cn/gsa) under accession number CRA042833.

## SUPPLEMENTAL MATERIAL

Supplemental material aem.00819-26-s0001.pdf Fig. S1 to S17; Tables S1 to S7.

ASM does not own the copyrights to Supplemental Material that may be linked to, or accessed through, an article. The authors have granted ASM a non-exclusive, world-wide license to publish the Supplemental Material files. Please contact the corresponding author directly for reuse.
