# Supplementary material for: Marine antifouling biocide 4,5-dichloro-2-n-octyl-4-isothiazolin-3-one disrupts sediment microbiome structure and function: insights from absolute quantification and enzyme activity dynamics
Source: Appl Environ Microbiol. 2026 Jul 24;92(8):e00819-26. doi: 10.1128/aem.00819-26 (PMC13488314; doi:10.1128/aem.00819-26)
Supplement: Supplementary file 1 [file aem.00819-26-s0001.pdf]

Supplemental materials for

Marine antifouling biocide 4,5-dichloro-2-n-octyl-4-isothiazolin-3-one disrupts sediment microbiome structure and function: Insights from absolute quantification and enzyme activity dynamics

Jiaxin Zhang, Libing Cai, Lin Wang, Lianhui Zhang, Nan Meng, Andong Chen, Qiao Ma*

Institute of Environmental Systems Biology, College of Environmental Science and Engineering, Dalian Maritime University, Dalian 116026, China

*Corresponding author. *E-mail address*: xiaoma0556@dlmu.edu.cn (Qiao Ma)

**Figures**	**17**
**Tables**	**7**

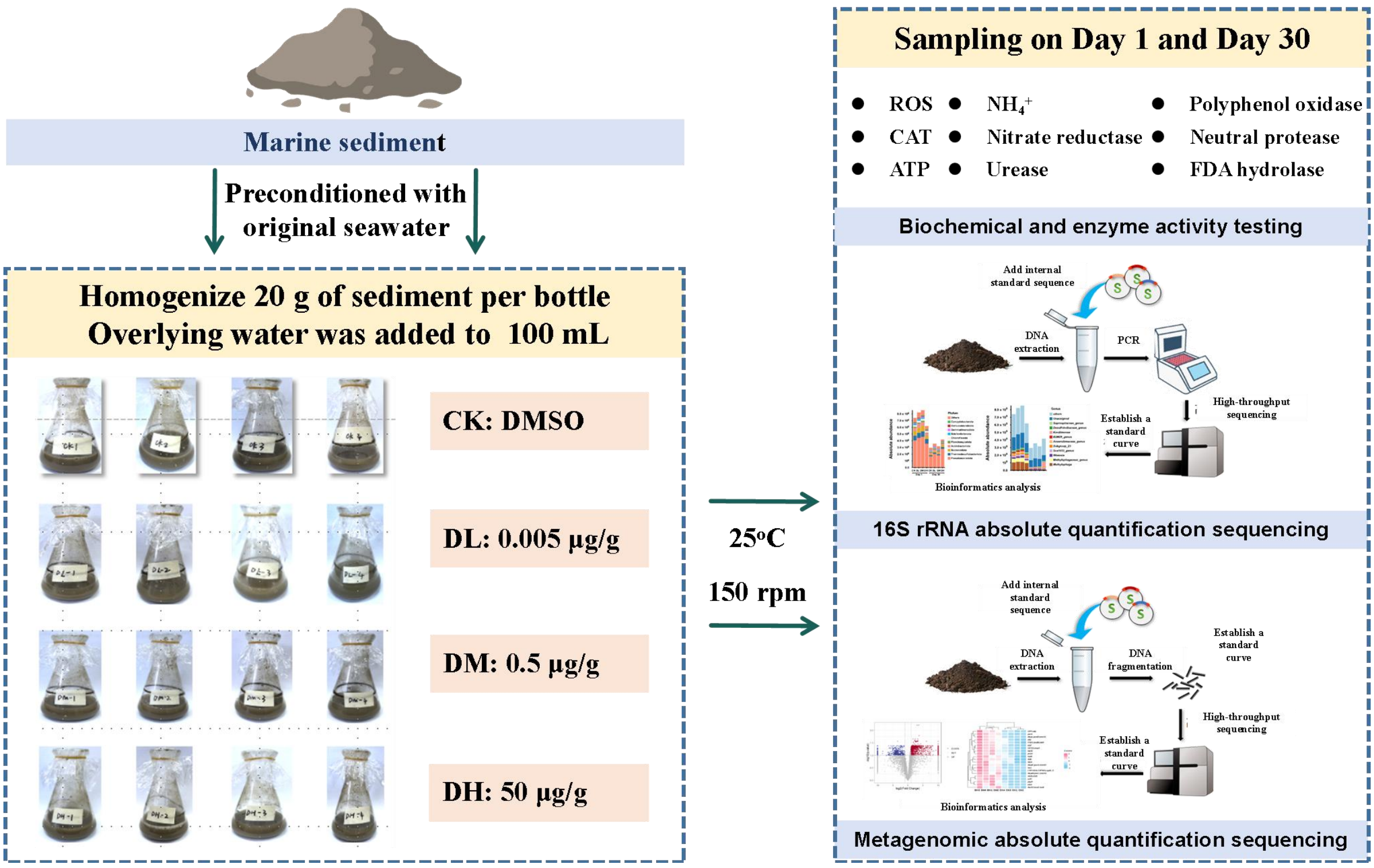

Fig. S1 Experimental design and analytical workflow of the study. Sediments were exposed to a DCOIT gradient (0-50 μg/g) for 30 days. The ecological and functional responses were characterized using a combination of enzymatic assays, absolute quantitative 16S rRNA sequencing, and metagenomics

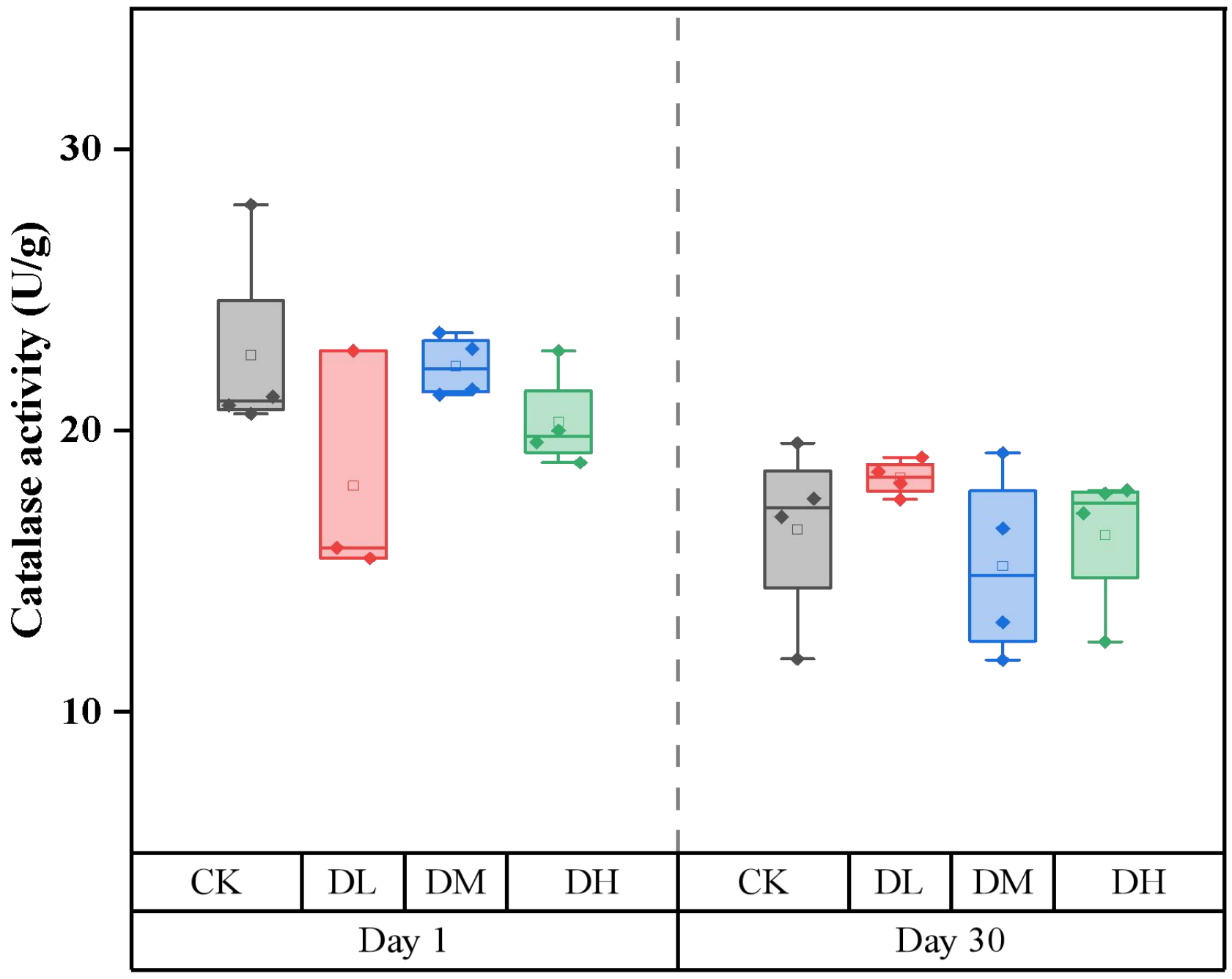

Fig. S2 CAT activity in different groups on Day 1 and Day 30

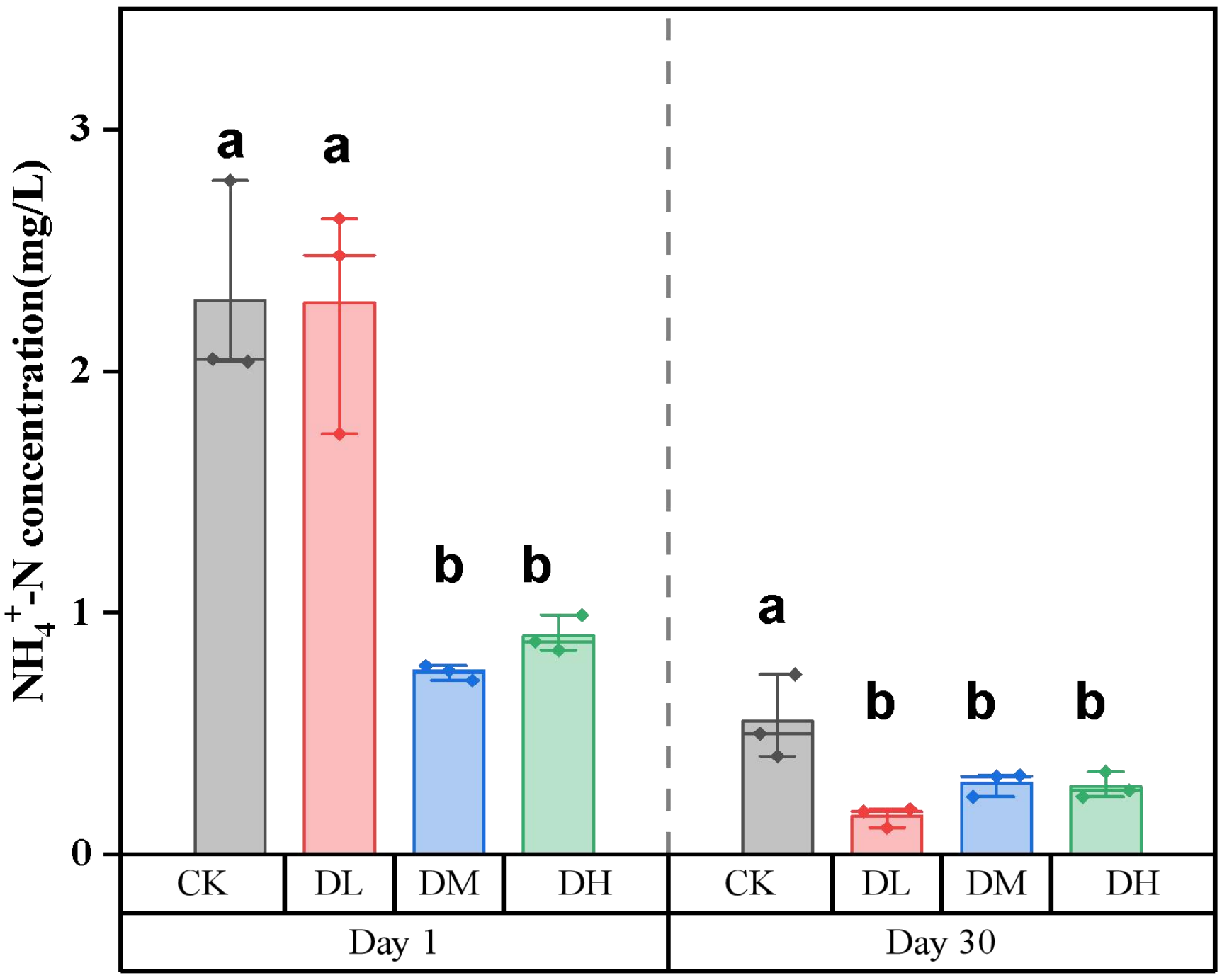

Fig. S3 NH_4^+ concentrations in different groups on Day 1 and Day 30

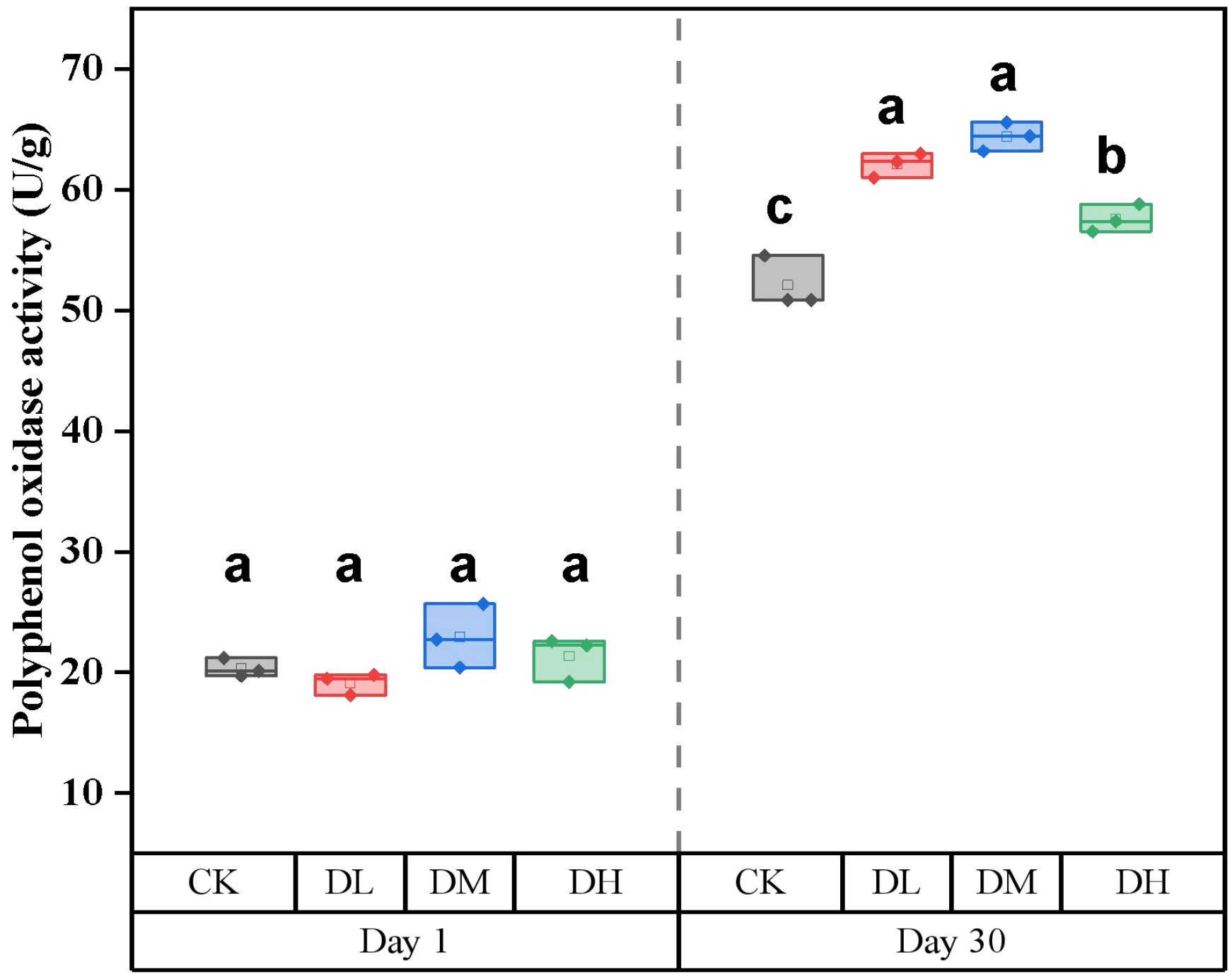

Fig. S4 PPO activity in different groups on Day 1 and Day 30

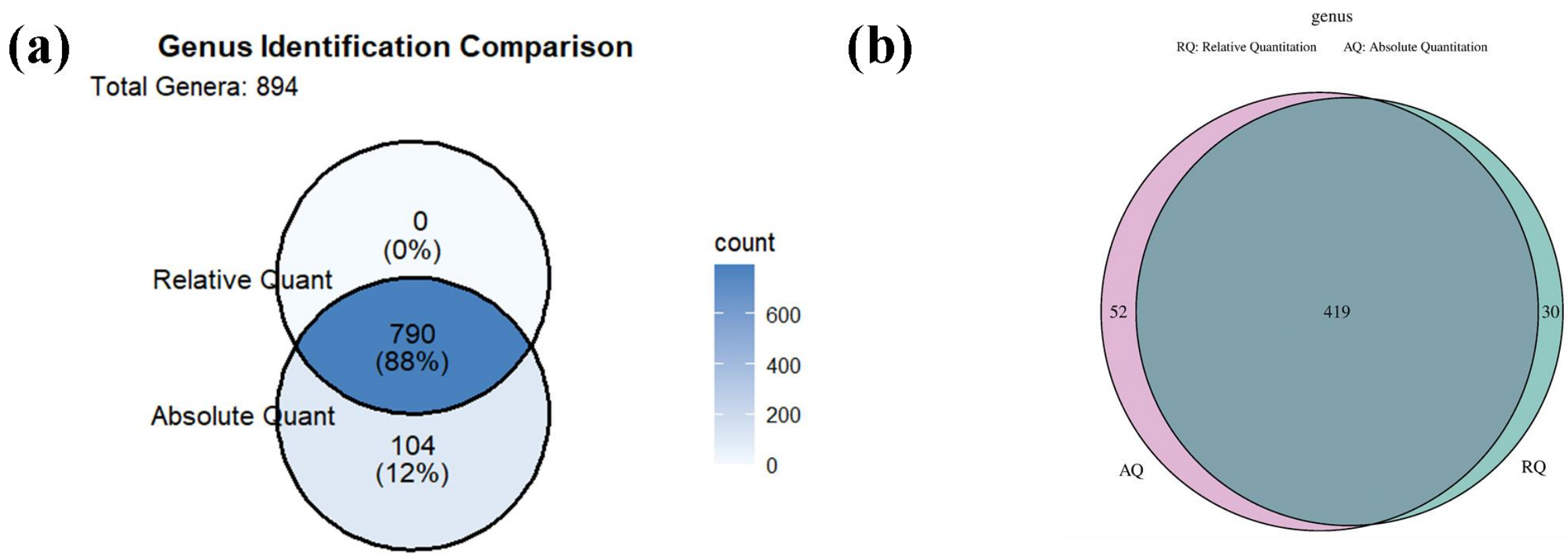

Fig. S5 Comparison of genus-level taxa identified by absolute- and relative-abundance-based profiling. (a) Total number of genera and (b) number of differentially abundant genera.

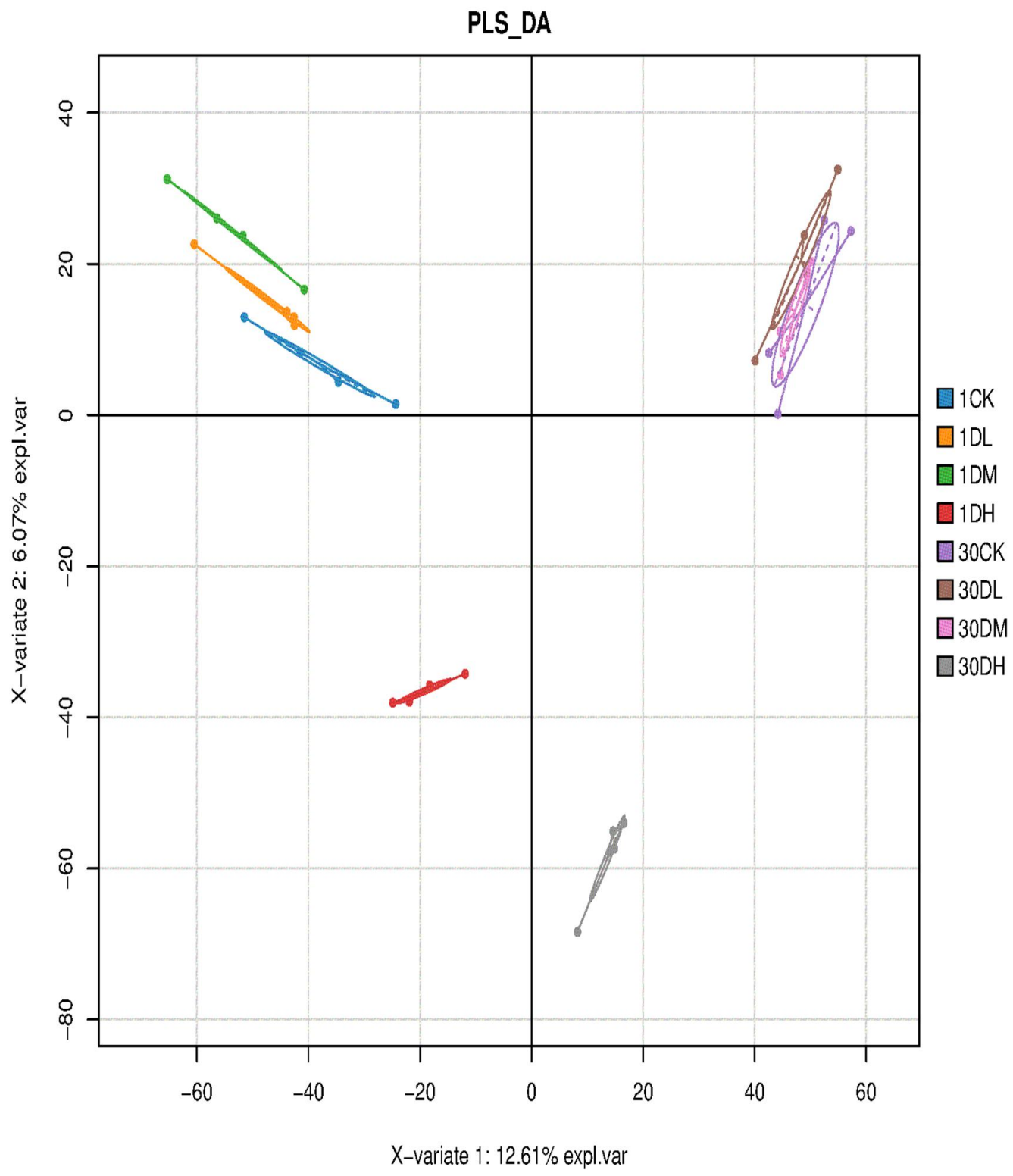

Fig. S6 Partial least squares discriminant analysis of bacterial community structure

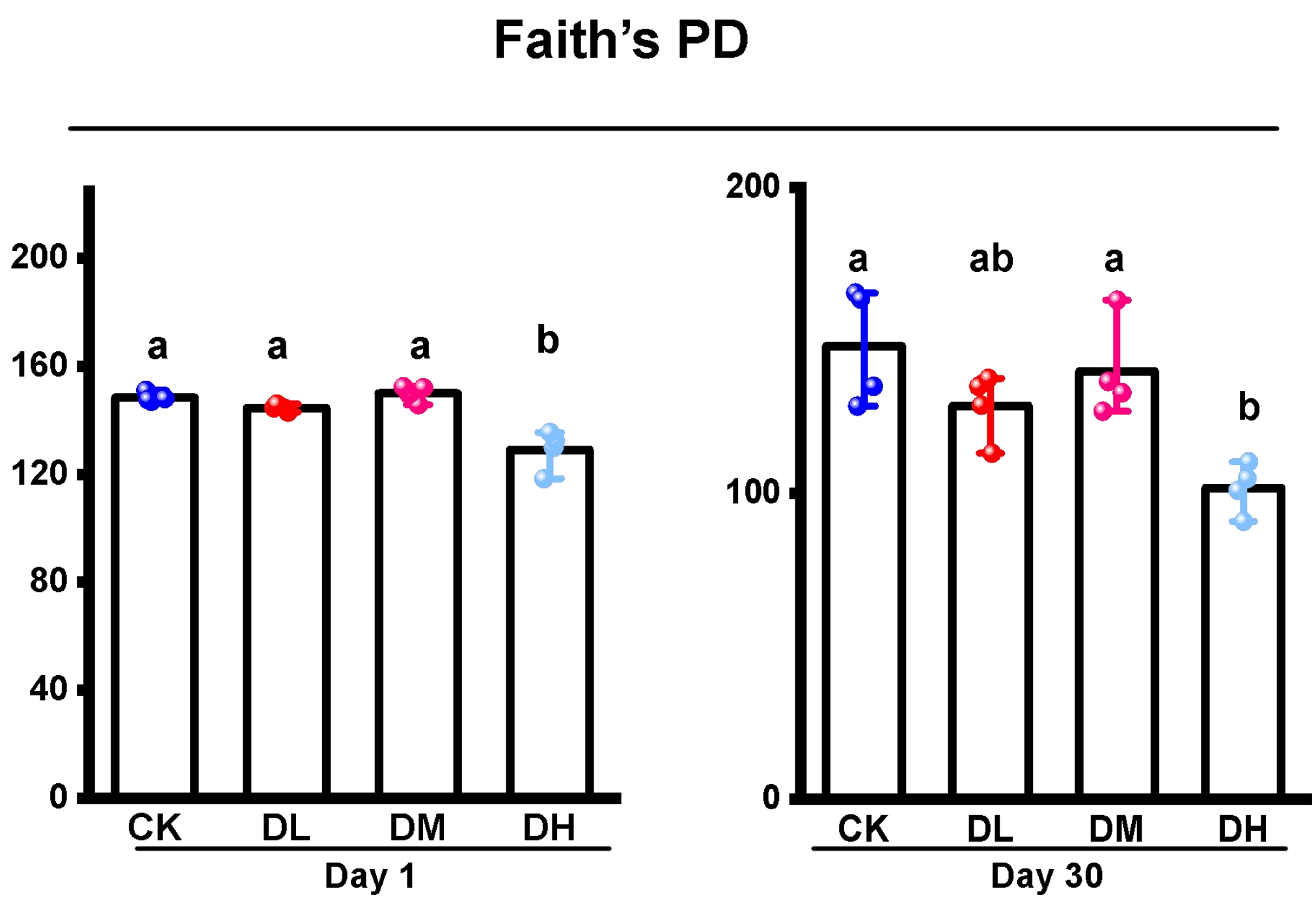

Fig. S7 Effects of DCOIT on the Faith's PD of the marine sediment microbial community

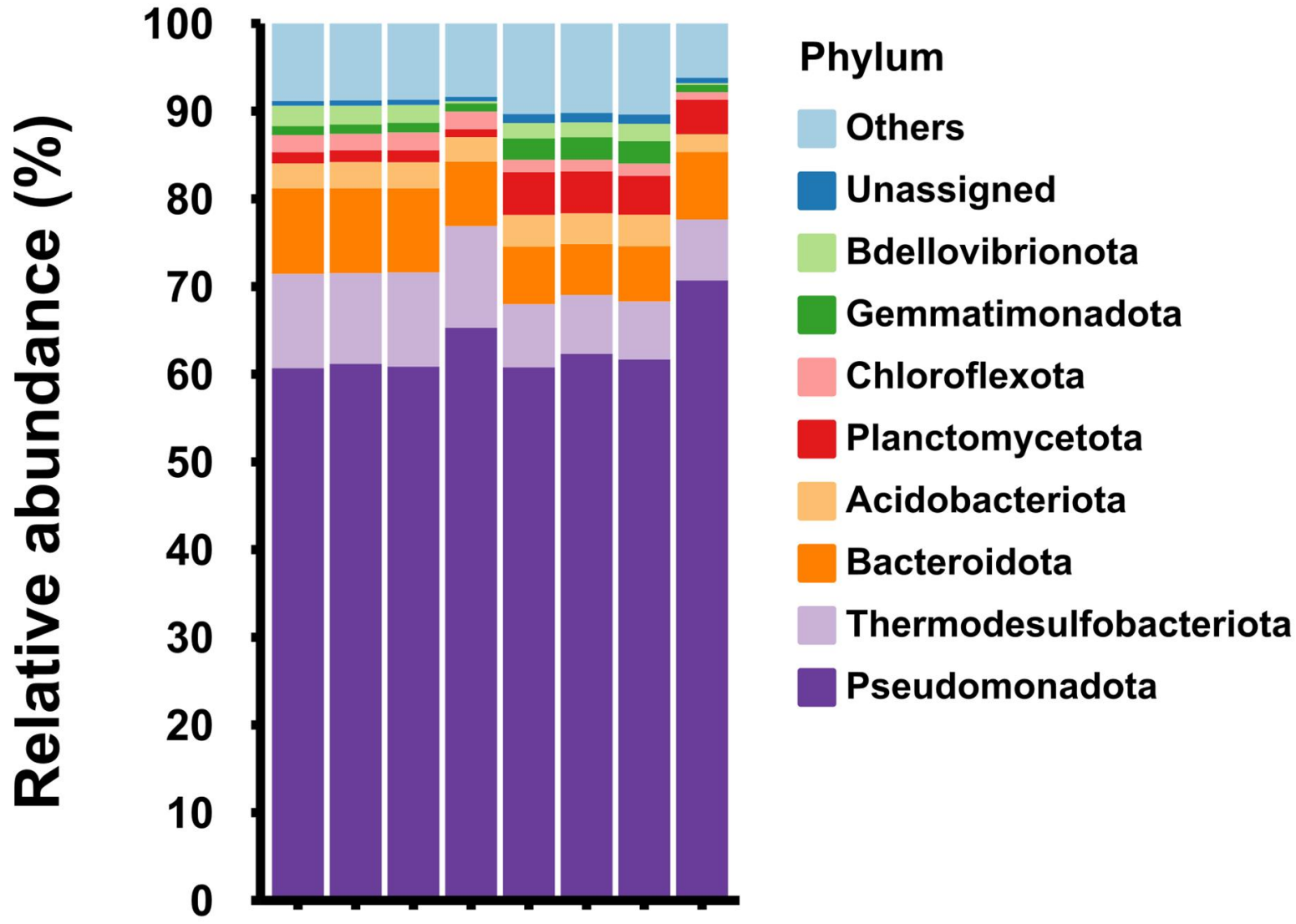

Fig. S8 Shifts in bacterial community composition at the phylum level based on relative-abundance profiling

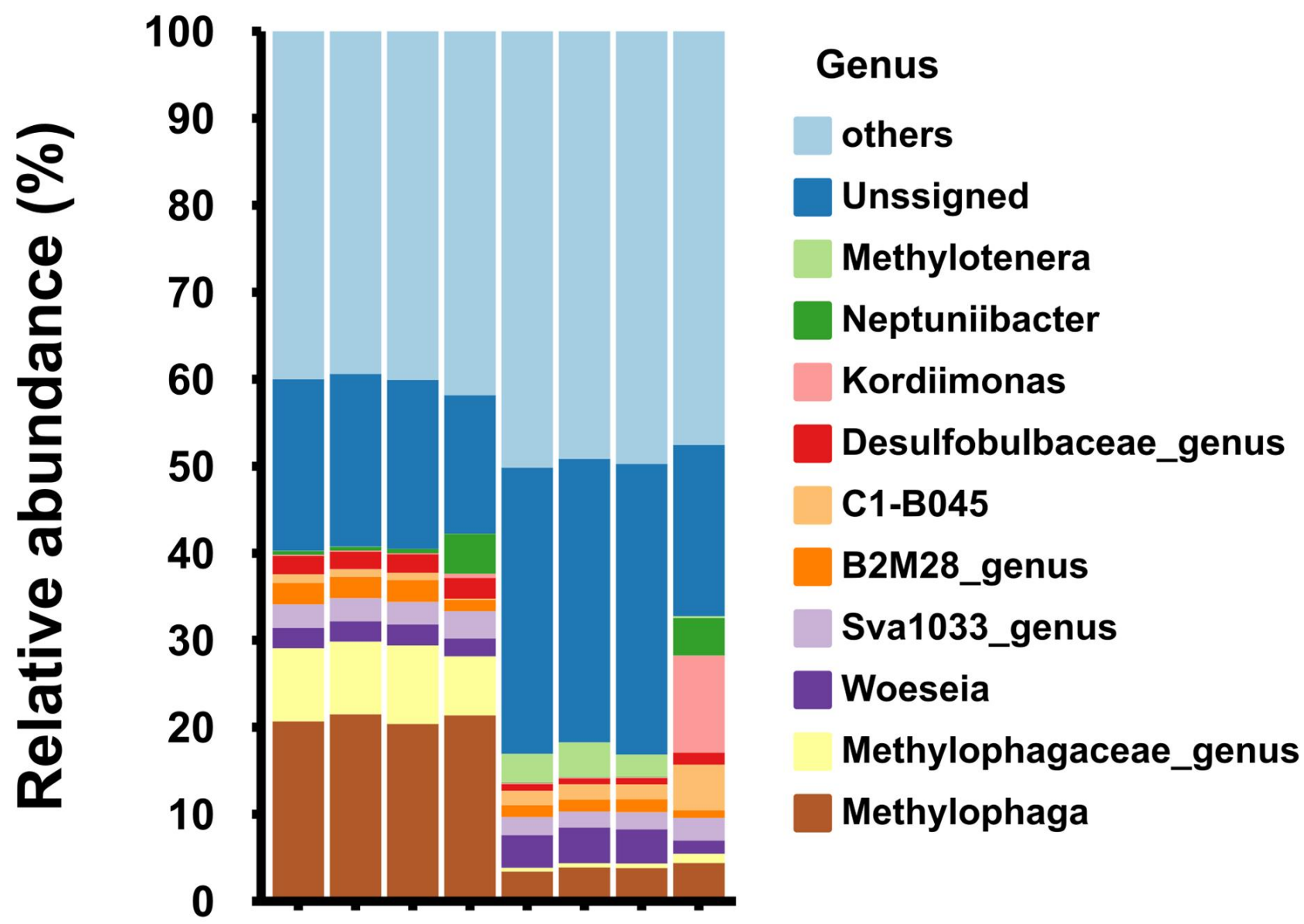

Fig. S9 Shifts in bacterial community composition at the genus level based on relative-abundance profiling

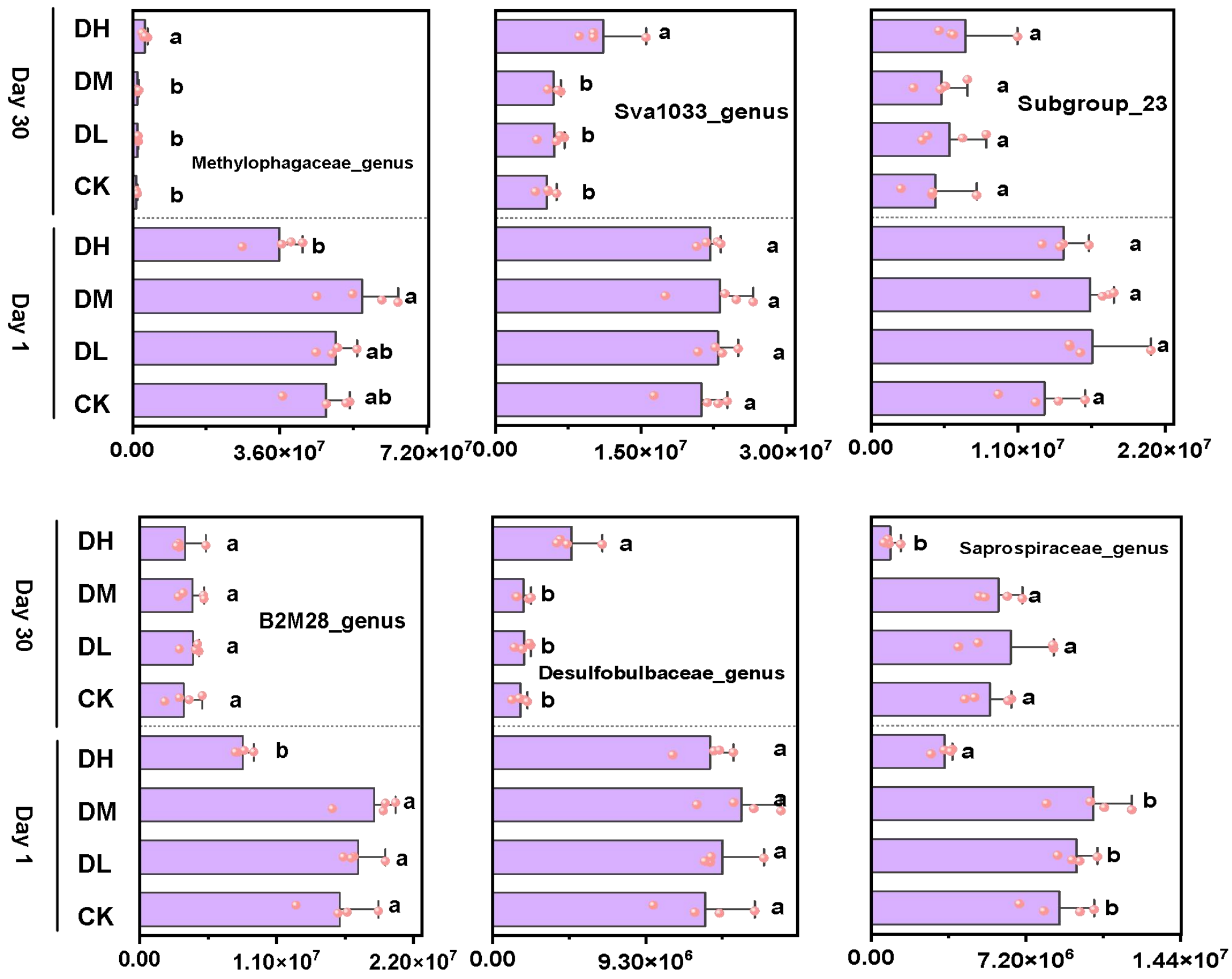

Fig. S10 Absolute abundances of selected dominant microbial genera

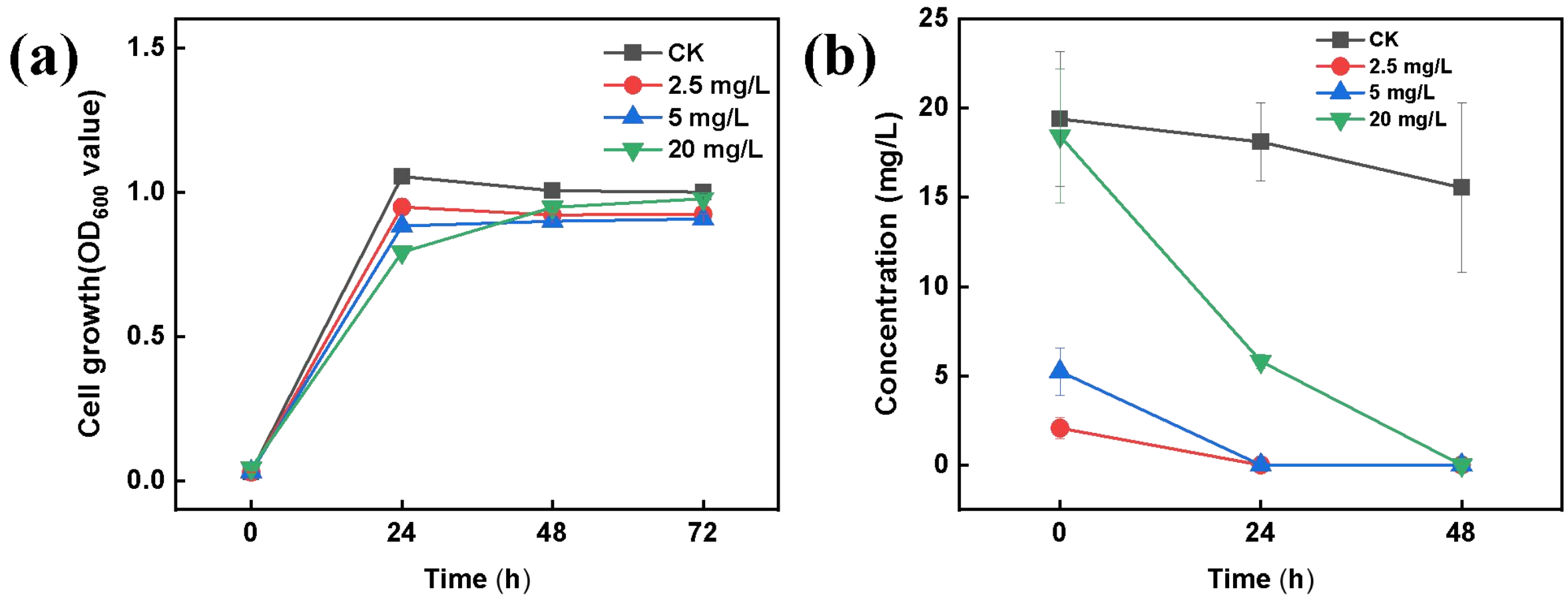

Fig. S11 Growth of the acclimated bacterial consortium and DCOIT degradation during incubation. (a) Growth curves and (b) DCOIT degradation curves

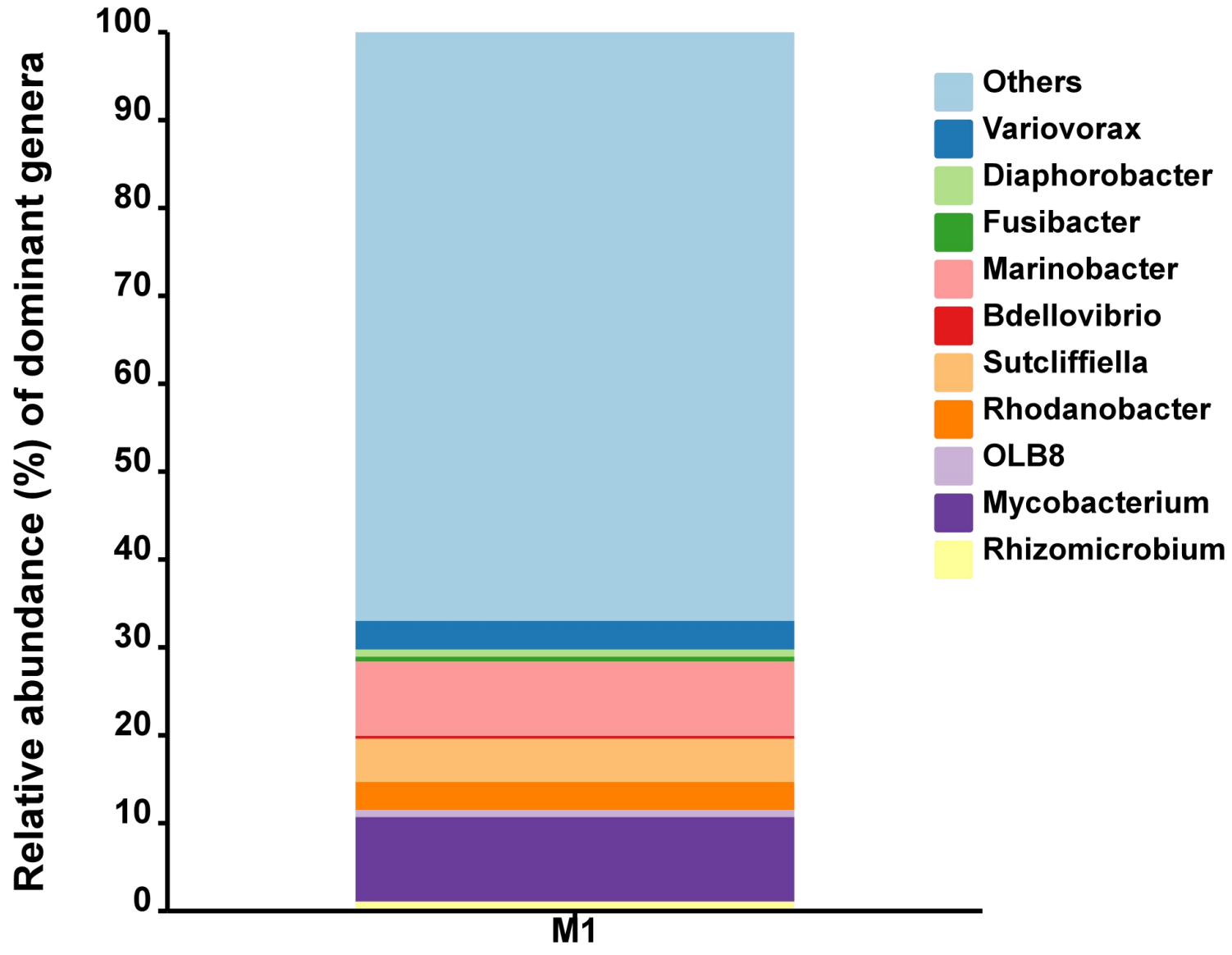

Fig. S12 Relative abundance of dominant bacterial genera in the acclimated consortium

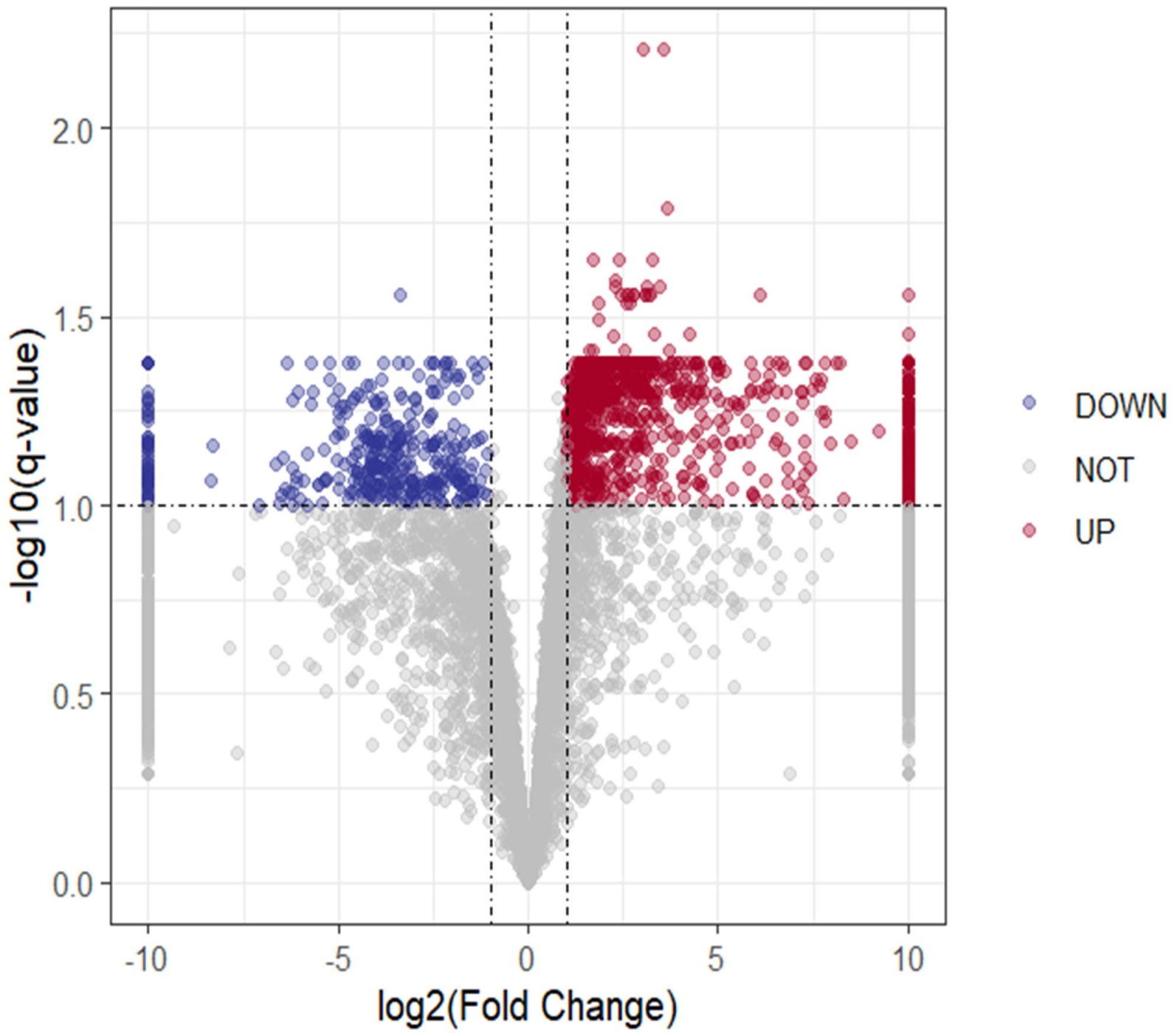

Fig. S13 Volcano plot of differentially abundant KOs between the DH and CK groups

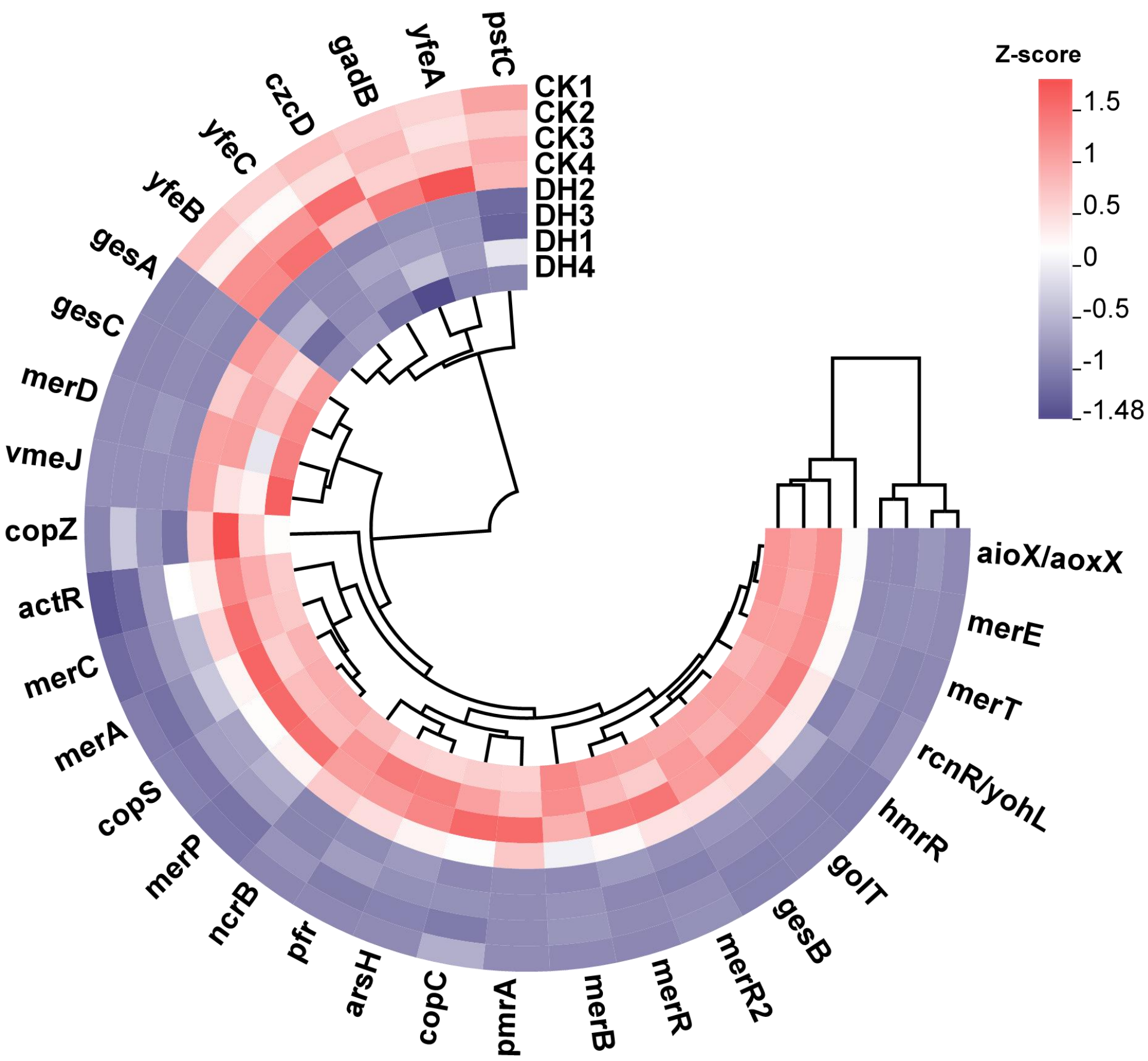

Fig. S14 Heatmap of differentially abundant BacMet-annotated resistance genes

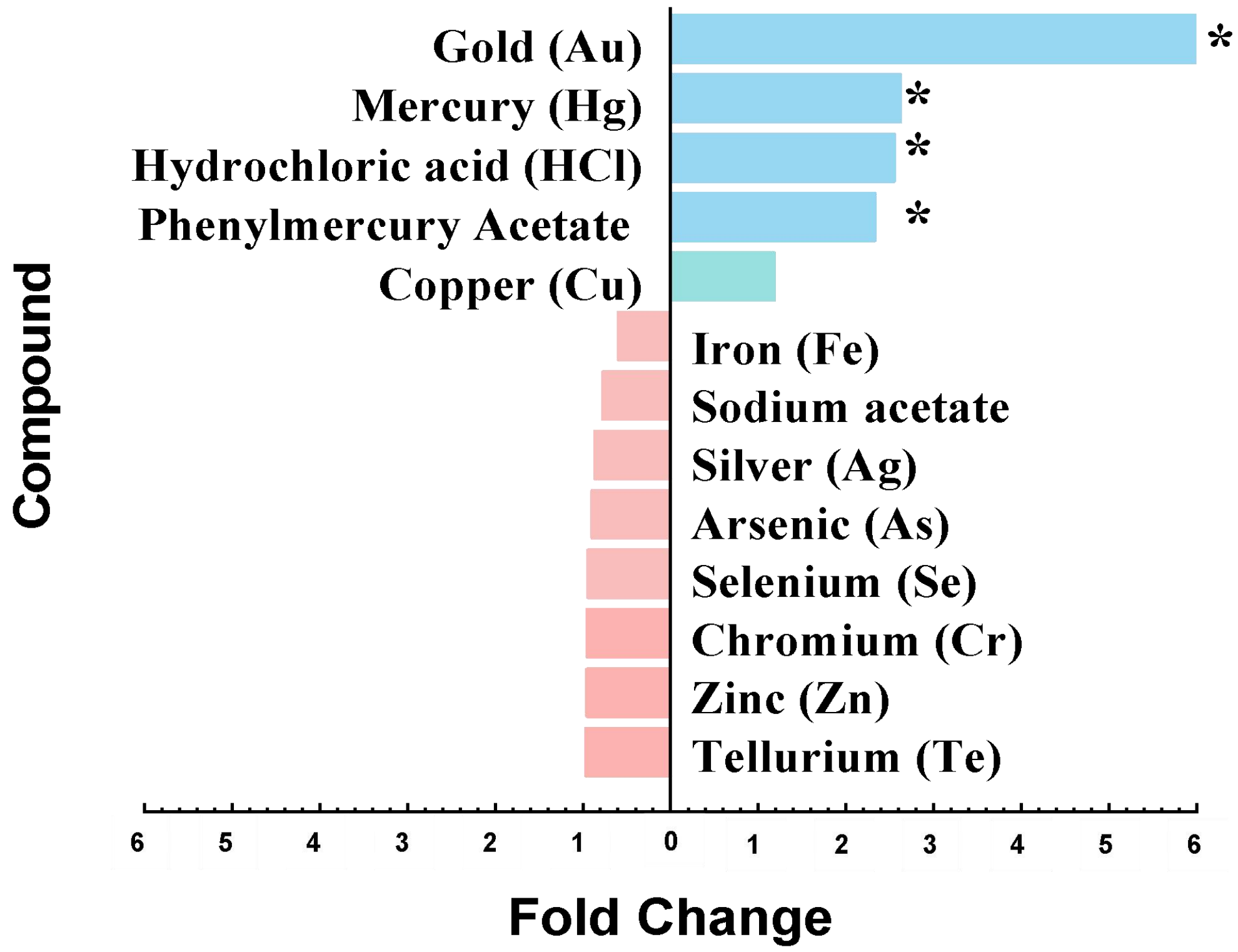

Fig. S15 Fold changes of major BacMet resistance categories in the DH group relative to the CK group. Asterisks indicate statistically significant differences

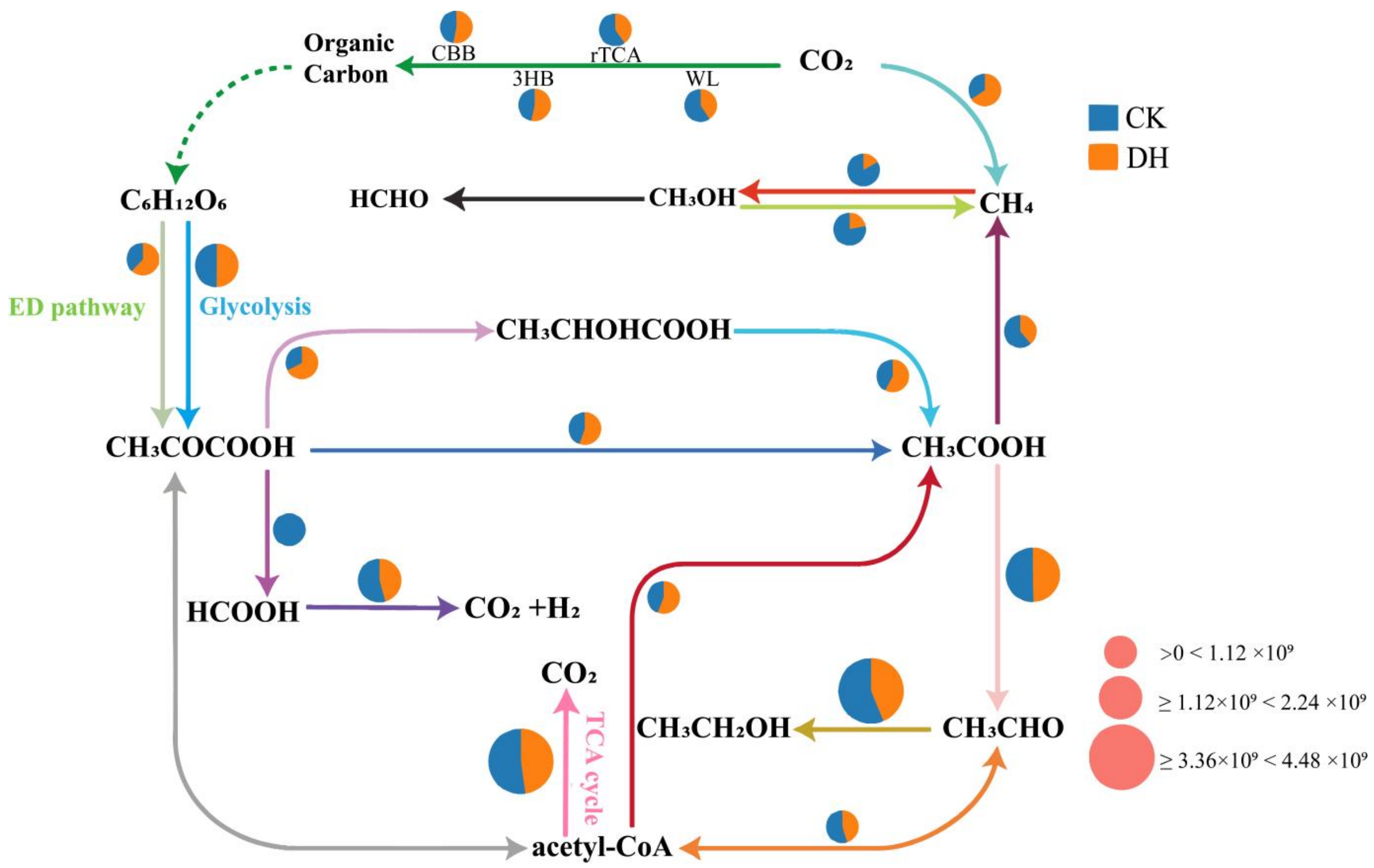

Fig. S16 Metagenomic potential of carbon cycling under DCOIT stress

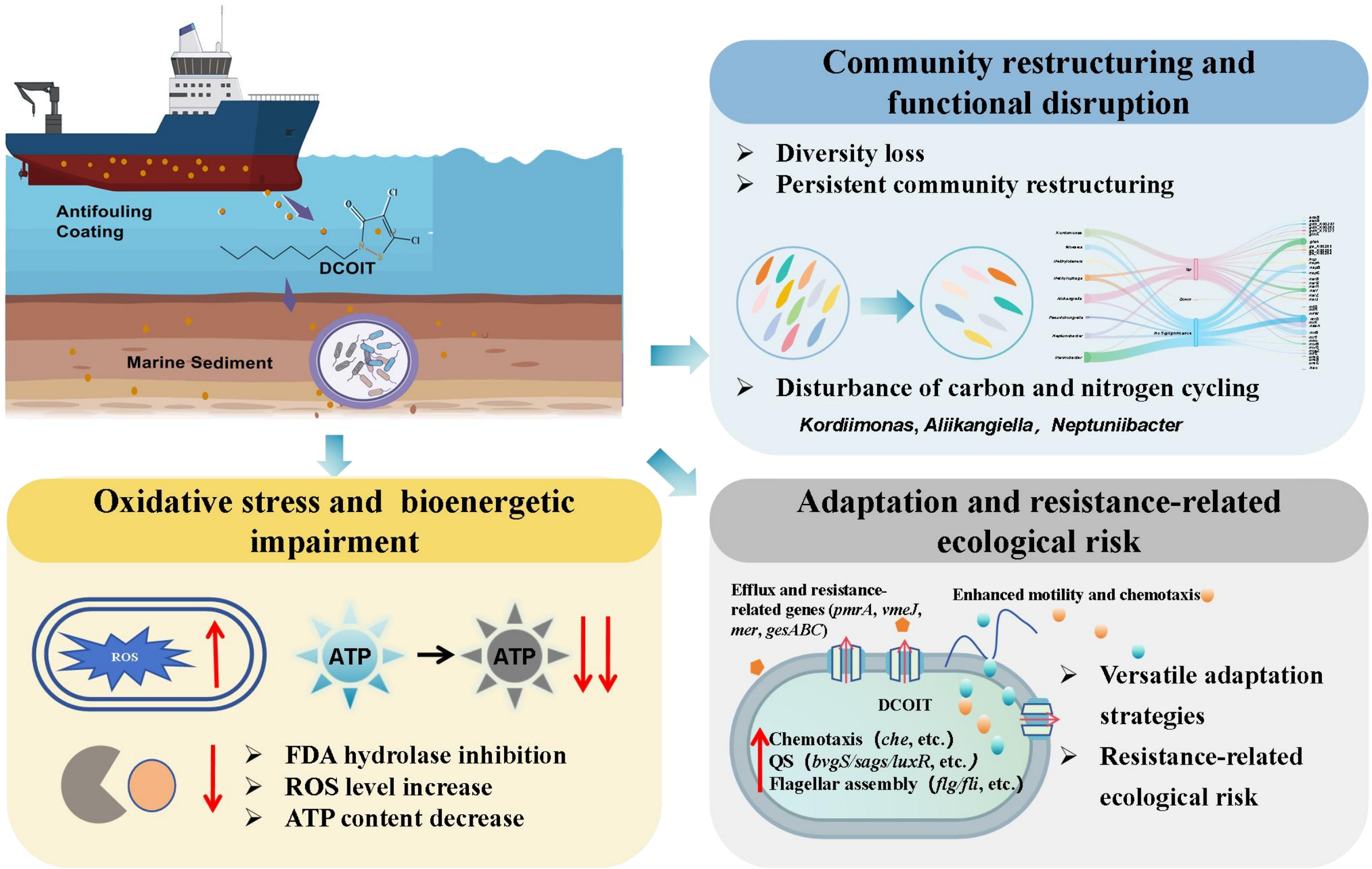

Fig. S17 Integrated impacts of DCOIT on marine microbial community

Table S1 Total microbial absolute abundance in different treatment groups

Samples	Abundance	Samples	Abundance	Samples	Abundance	Samples	Abundance
1CK1	77734563	1DM1	689757432	30CK1	148491547	30DM1	224251051
1CK2	876117333	1DM2	845750446	30CK2	346758596	30DM2	363143868
1CK3	752007952	1DM3	909048322	30CK3	263252236	30DM3	285524105
1CK4	562350828	1DM4	977246098	30CK4	424388321	30DM4	391646006
1DL1	802622509	1DH1	621716076	30DL1	389351480	30DH1	364745276
1DL2	784797301	1DH2	658196565	30DL2	250117476	30DH2	562601749
1DL3	945198668	1DH3	526024003	30DL3	452669047	30DH3	382098394
1DL4	755461571	1DH4	670232695	30DL4	334620890	30DH4	362078660

Table S2 The alpha diversity of communities in different treatment groups

Samples	Observed	Shannon	Simpson	Faith's PD
1CK	2689.00	6.14	0.0180	148.49
1DL	2648.75	6.19	0.0169	144.57
1DM	2690.75	6.24	0.0152	150.00
1DH	2137.25	5.86	0.0224	129.09
30CK	2860.50	6.56	0.0061	148.11
30DL	2286.50	6.44	0.0065	128.62
30DM	2657.25	6.43	0.0070	139.86
30DH	1674.50	5.78	0.0123	101.73

Table S3 Fold changes and taxonomic contributions of key nitrogen-cycling genes under DCOIT exposure

Taxon	Gene	Functional pathway	Fold change
Kordiimonas	*asnB*	Ammonia assimilation	14.14
Kordiimonas	*narG*	Denitrification	5.60
Kordiimonas	*narH*	Denitrification	6.95
Kordiimonas	*narI*	Denitrification	215.93
Kordiimonas	*nirB*	Dissimilatory nitrate reduction to ammonium	14.94
Kordiimonas	*nirD*	Dissimilatory nitrate reduction to ammonium	254.62
Kordiimonas	*nirK*	Denitrification	17.71
Kordiimonas	*nirS*	Denitrification	11.19
Aliikangiella	*asnB*	Ammonia assimilation	7.41
Aliikangiella	*hcp*	Others	9.37
Aliikangiella	*napA*	Denitrification	103.73
Aliikangiella	*napC*	Denitrification	98.13
Aliikangiella	*narG*	Denitrification	15.74
Aliikangiella	*narH*	Denitrification	6.98
Aliikangiella	*narI*	Denitrification	91.82
Aliikangiella	*narJ*	Denitrification	177.68
Aliikangiella	*narZ*	Denitrification	6.60
Aliikangiella	*nirB*	Assimilatory nitrate reduction to ammonium	640.28
Aliikangiella	*nirK*	Denitrification	28.91
Aliikangiella	*nirS*	Denitrification	13.27
Neptuniibacter	*nasA*	Assimilatory nitrate reduction to ammonium	473.76
Neptuniibacter	*nirB*	Dissimilatory nitrate reduction to ammonium	12.43
Neptuniibacter	*nirK*	Denitrification	3.13
Neptuniibacter	*norB*	Denitrification	42.30

Table S4 Taxonomic contributions of microbial taxa to *amoABC* genes and their associated fold changes

Taxon	Gene	Fold change
Nitrosomonas	*amoA_B*	0.87
Nitrosomonas	*amoB_B*	1.34
Nitrosomonas	*amoC_B*	1.21
Nitrosopumilus	*amoA_A*	0.09
Nitrosopumilus	*amoB_A*	0.07
Nitrosopumilus	*amoC_A*	0.07
Nitrosospira	*amoA_B*	0.12
Nitrosospira	*amoB_B*	0.08
Nitrosospira	*amoC_B*	0.00
Nitrosovibrio	*amoC_B*	0.05
Unassigned	*amoB_B*	0.12
unclassified_Nitrosomonadaceae	*amoA_B*	0.06
unclassified_Nitrosomonadaceae	*amoC_B*	0.20

Table S5 Genomic characteristics and quality assessment of metagenome-assembled genomes (MAGs) recovered from microbial communities

Bin	Completeness (%)	Contamination (%)	Genome size (bp)	GC (%)
DCK3_30d.metabat2.bin.35	98.7	0.65	3129276	58.6
DH1_30d.metabat2.bin.21	94.07	0.76	3318208	55.4
DH3_30d.maxbin2.bin.0	96.92	0.8	3011826	55.3
DCK2_30d.metabat2.bin.31	96.23	0.93	2680554	41.4
DH4_30d.metabat2.bin.28	94.61	1.07	2835099	50.7
DCK2_30d.concoct.bin.17	75.98	1.16	2611794	62.6
DH1_30d.concoct.bin.29	96.38	1.22	2958730	55.6
DH4_30d.concoct.bin.50	95.93	1.3	2462341	55.5
DH4_30d.metabat2.bin.25	98.33	1.3	2737762	59.1
DH3_30d.concoct.bin.5	96.69	1.43	2248464	44.1
DH1_30d.concoct.bin.32	94.62	1.71	6115069	69.3
DCK3_30d.concoct.bin.12	90.09	1.74	1967753	54.6
DH3_30d.concoct.bin.7	76.88	1.86	2837427	50.3
DH2_30d.concoct.bin.93	95.41	1.94	3758958	45.2
DCK3_30d.metabat2.bin.30	91.95	1.95	2244949	63.1
DCK1_30d.concoct.bin.37	78.63	2.14	2217313	42.4
DCK3_30d.maxbin2.bin.2	94.02	2.28	2256463	41.2
DH3_30d.metabat2.bin.20	99.43	2.29	4987243	44.1
DH2_30d.metabat2.bin.44	90.13	2.59	2448189	58.3
DH4_30d.metabat2.bin.38	100	2.62	4021367	49.3
DCK2_30d.metabat2.bin.20	94.47	2.97	2996503	37.5
DH1_30d.metabat2.bin.36	95.24	3.24	3197241	47.6
DCK4_30d.metabat2.bin.11	95.95	3.26	2701121	41.4
DH2_30d.concoct.bin.61	89.62	3.35	4132616	68.6
DH2_30d.metabat2.bin.28	94.32	3.62	6629052	68.7
DH1_30d.concoct.bin.40	83.82	3.7	3003401	33.6
DCK3_30d.metabat2.bin.36	92.47	4.18	1951100	49.9
DH1_30d.metabat2.bin.7	92.04	5.25	2901525	55.3
DH2_30d.concoct.bin.51	90.21	5.65	9693895	69
DH3_30d.metabat2.bin.21	96.99	6.67	3299294	54.5
DCK2_30d.metabat2.bin.23	79.62	6.77	2882013	38.9
DH4_30d.maxbin2.bin.17	90.12	7.38	3594292	56.7
DCK1_30d.metabat2.bin.33	91.25	8.17	2694923	55.6
DH1_30d.concoct.bin.49	80	8.61	2150070	51.9
DCK4_30d.maxbin2.bin.17	81.01	8.81	3282172	50.8
DCK2_30d.maxbin2.bin.18	77.71	9.29	3682588	51.5

Table S6 Taxonomic contributions of microbial taxa to *merA* genes and their associated fold changes

Taxon	Gene	Fold change
Unassigned	*merA*	1.40
Porticoccus	*merA*	13.94
unclassified_Gammaproteobacteria	*merA*	3.08
Marinobacter	*merA*	48.16
Gimibacter	*merA*	38.44
Neptuniibacter	*merA*	6.99
unclassified_Alphaproteobacteria	*merA*	78.63
Tistrella	*merA*	1.13
Pelagibius	*merA*	0.63
Pseudaestuariivita	*merA*	0.32

Table S7 Taxonomic contributions of microbial taxa to *gesABC* genes and their associated fold changes

Taxon	Gene	Fold change
Alcanivorax	*gesB*	2.76
Eilatimonas	*gesB*	1.50
Erythrobacter	*gesB*	38.99
Marinobacter	*gesA*	110.01
Marinobacter	*gesB*	27.16
Marinobacter	*gesC*	214.82
Neptuniibacter	*gesB*	281.46
Ponticaulis	*gesB*	26.63
Shewanella	*gesB*	ND in CK
Unassigned	*gesB*	1.31
unclassified_Thalassospiraceae	*gesB*	0.23
